# Highly-purified rapidly expanding clones, RECs, are superior for functional-mitochondrial transfer

**DOI:** 10.1186/s13287-023-03274-y

**Published:** 2023-03-16

**Authors:** Jiahao Yang, Lu Liu, Yasuaki Oda, Keisuke Wada, Mako Ago, Shinichiro Matsuda, Miho Hattori, Tsukimi Goto, Yuki Kawashima, Yumi Matsuzaki, Takeshi Taketani

**Affiliations:** 1 Department of Pediatrics, Faculty of Medicine, Shimane University, 89-1, Enya, Izumo, Shimane 693-8501 Japan; 2 Department of Medical Oncology, Shimane University Hospital, Izumo, Shimane Japan; 3 Department of Life Science, Faculty of Medicine, Shimane University, Izumo, Shimane Japan; 4 Faculty of Nursing, Inner Mongolia Medical University, Hohhot, Inner Mongolia China

**Keywords:** Mesenchymal stem cells (MSCs), Rapidly expanding clones (RECs), Mitochondrial transfer, Mitochondrial dysfunction

## Abstract

**Background:**

Mitochondrial dysfunction caused by mutations in mitochondrial DNA (mtDNA) or nuclear DNA, which codes for mitochondrial components, are known to be associated with various genetic and congenital disorders. These mitochondrial disorders not only impair energy production but also affect mitochondrial functions and have no effective treatment. Mesenchymal stem cells (MSCs) are known to migrate to damaged sites and carry out mitochondrial transfer. MSCs grown using conventional culture methods exhibit heterogeneous cellular characteristics. In contrast, highly purified MSCs, namely the rapidly expanding clones (RECs) isolated by single-cell sorting, display uniform MSCs functionality. Therefore, we examined the differences between RECs and MSCs to assess the efficacy of mitochondrial transfer.

**Methods:**

We established mitochondria-deficient cell lines (ρ^0^ A549 and ρ^0^ HeLa cell lines) using ethidium bromide. Mitochondrial transfer from RECs/MSCs to ρ^0^ cells was confirmed by PCR and flow cytometry analysis. We examined several mitochondrial functions including ATP, reactive oxygen species, mitochondrial membrane potential, and oxygen consumption rate (OCR). The route of mitochondrial transfer was identified using inhibition assays for microtubules/tunneling nanotubes, gap junctions, or microvesicles using transwell assay and molecular inhibitors.

**Results:**

Co-culture of ρ^0^ cells with MSCs or RECs led to restoration of the mtDNA content. RECs transferred more mitochondria to ρ^0^ cells compared to that by MSCs. The recovery of mitochondrial function, including ATP, OCR, mitochondrial membrane potential, and mitochondrial swelling in ρ^0^ cells co-cultured with RECs was superior than that in cells co-cultured with MSCs. Inhibition assays for each pathway revealed that RECs were sensitive to endocytosis inhibitor, dynasore.

**Conclusions:**

RECs might serve as a potential therapeutic strategy for diseases linked to mitochondrial dysfunction by donating healthy mitochondria.

**Supplementary Information:**

The online version contains supplementary material available at 10.1186/s13287-023-03274-y.

## Background

Mitochondria drive cellular metabolism and are crucial regulators of life and death [1, 2]. They are essential for processes such as ATP synthesis, oxidative phosphorylation (OXPHOS), and cellular signal transduction [3]. Several mitochondria-specific proteins, rRNAs, and tRNAs are encoded by the mitochondrial genome (mtDNA) [4]. However, mitochondrial damage can occur due to the absence of protective histones, lack of post-injury repair capacity, proximity to the cell's main source of reactive oxygen species (ROS), and its susceptibility to damage. In a self-sustaining damage cycle, mitochondrial ROS (mtROS) damages mtDNA, hence producing less effective mitochondria, which in turn produce more mtROS [5, 6]. Therefore, therapies that attempt to reduce mitochondrial malfunction and maintain mtDNA stability are crucial.

**Fig. 1:**
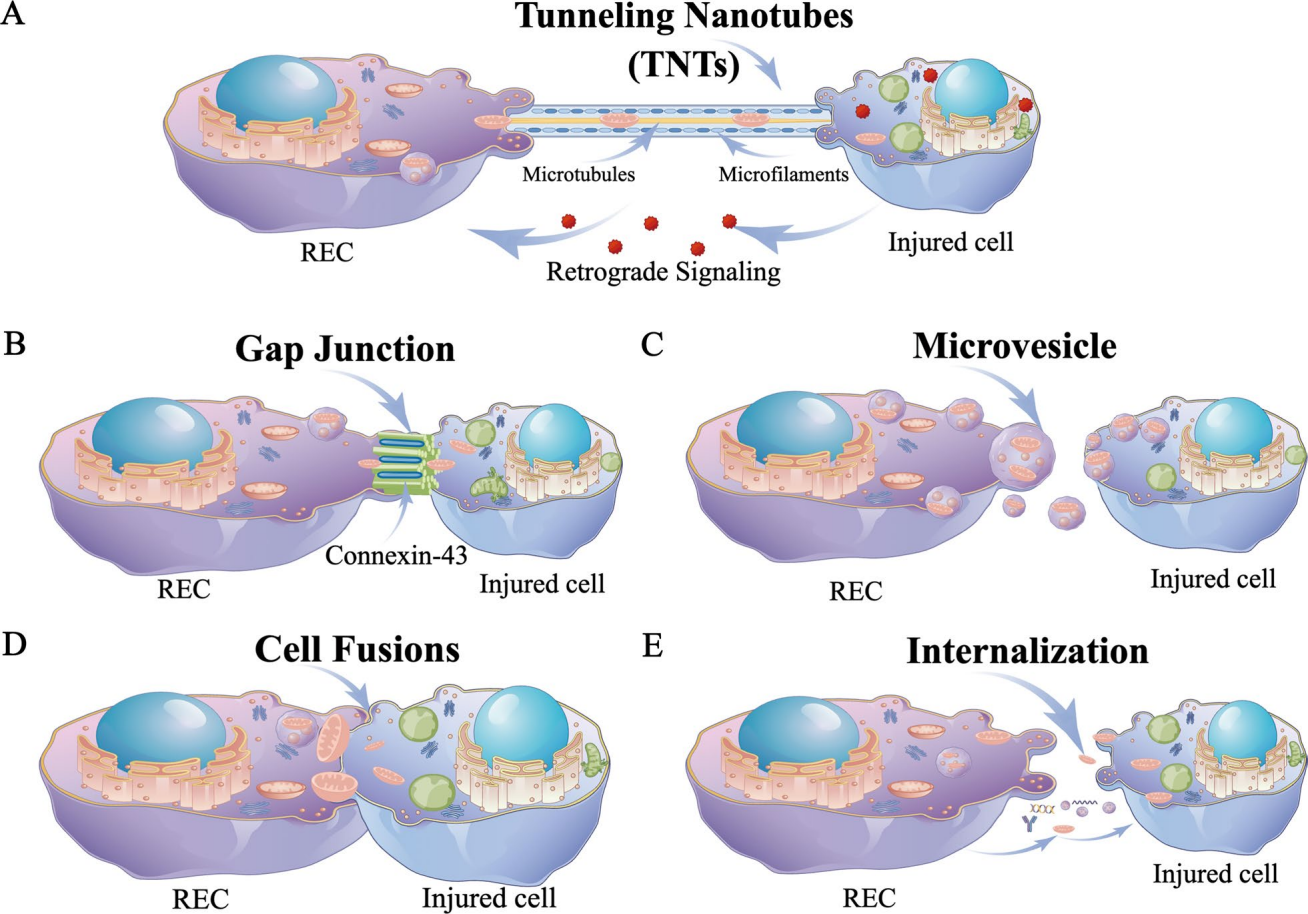
Schematic diagram demonstrating routes of mitochondrial transfer from RECs to recipient cells. **A** TNTs are membranous tubules that extend from the plasma membrane and measure 50–1500 nm in diameter. It is the most well-known route for mitochondrial transfer between cells. **B** Connexin 43 (CX43) is a gap junction protein that regulates a cell connection channel and facilitates organelle exchange or cell migration. CX43 is an essential regulator of mitochondrial transfer. **C** Another pathway for mitochondrial transfer has been observed as microvesicles generated by blebbing the cellular plasma membrane. **D** Cell fusion, possible route for mitochondrial transfer.** E** Without carriers, free mitochondria can be extruded or internalized, suggesting a plausible mechanism for intercellular mitochondrial transfer. Make figure by Figdraw

There is limited knowledge about the mechanisms governing mitochondrial transport between cells [7]. The integration of mitochondrial genes or the mitochondria themselves into the recipient cell occurs during their transfer. The host's biological energy level may be significantly altered by this phenomenon, along with changes in cell differentiation, inflammatory reactions, cell survival, and even treatment resistance. Transcellular mitochondrial transport has been demonstrated to be possible in a number of structures, including tunneling nanotubes (TNTs) [8–10], connexin 43 (Cx43) gap junctions (GJs) [11, 12], microvesicles (MVs) [9, 13, 14], cell fusion [15, 16], internalization [17, 18] (Fig. 1A–E).

Researchers have been interested in exogenous replacement of damaged mitochondria to prevent cell death [19]. In 2006, it was revealed that mitochondria from human mesenchymal stem cells (MSCs) might be transported into defective cells through TNTs [20]. Furthermore, MSCs isolated from bone marrow (BM) [9, 19, 21, 22] and fat [21, 23] is likely to repair the mitochondrial activity of the recipient cells by transferring their own mitochondria. However, the conventionally isolated bone marrow-derived MSCs (BMSCs) used in clinical research has shown different ability for cellular proliferation and differentiation and even contradictory results because these BMSCs always contain undifferentiated cells leading to a heterogeneous cell population with inconsistent functions. Our previous work reported that rapidly expanding clones (RECs) were isolated as a single clone from CD90^high^/CD271^high^ population in bone marrow mononuclear cells [24]. This clonally expanded and ultra-purified BM-MSCs, RECs displays all the properties of MSCs, such as plastic adherence, differentiation capacity, and cell surface antigens, and does not exhibit lot-related variations in clinical applications.

RECs have a possibility to offer many potential benefits as transplantable cells for treating several disorders related to bone, heart, peripheral nerves, brain, and other organs [24, 25]. However, whether RECs can restore the bioenergetics of the cell remains unknown. Further, the health of the mitochondria transferred into mtDNA-deficient cells needs to be assessed. This study aimed to identify REC-dependent mitochondrial transport pathways and investigate whether this transfer restores cellular functions in mtDNA-deficient cells.

## Methods

### Cell culture, ρ^0^ cells and MSCs

Human cancer cell lines A549 and HeLa cells were purchased from RIKEN CELL BANK and cultured in Roswell Park Memorial Institute (RPMI) 1640 medium (Wako, Japan) supplemented with 1% penicillin–streptomycin and 10% fetal bovine serum (FBS; HyClone, USA) and were maintained at 37 ℃ in an atmosphere of 5% CO_2_-95% air. Rho 0 A549 (ρ^0^ A549) and Rho 0 HeLa (ρ^0^ HeLa) cells were established as previously described by culturing in the presence of ethidium bromide[26] (EtBr; Sigma, USA) (50 ng/mL), 1 × sodium pyruvate solution (Wako, Japan) (110 μg/mL), and uridine (Sigma, USA) (50 μg/mL) in RPMI 1640 supplemented with 10% FBS for more than 20 passages. Control parental A549 and HeLa cells were maintained in standard culture medium for the same period. We prepared three clones of BMSCs, MSC 1, 2, and 3 using BMSCs from Lonza (Basel, Switzerland). Human BMSCs were isolated as described previously [24] and cultured in DMEM/Ham’s F-12 medium (Wako, Japan) containing 15% FBS, 1% penicillin–streptomycin, and 10 ng/mL basic fibroblast growth factor (bFGF; Abcam; USA) (in 5% CO_2_, at 37 °C) until 80% confluent.

### Preparation of highly-purified MSCs (RECs)

Frozen GMP-compliant RECs provided by PuREC Co., Ltd. from Shimane University, were used in this study. RECs were isolated as a single clone from CD90^high^/CD271^high^ population in BM mononuclear cells. For the comparative analysis of RECs and normal human BMSCs, we prepared three clones of RECs: REC 1, REC 2, and REC 3. The cells were thawed in a warm bath at 37 °C prior to culturing and were directly used after thawing.

### Co-culture system (non-contact)

After labeling the mitochondria with 200 nM MitoTracker Green FM (Invitrogen, USA), MSCs and RECs were collected and inoculated into a cell culture insert (0.4 or 3 μm Transwell; Corning, USA). ρ^0^ A549 and ρ^0^ HeLa cell lines were labeled with 5 μL/mL of Vybrant™ DiD Cell-Labeling Solution (DiD) (Invitrogen, USA) and seeded in a 24-well plate, and co-cultured with MSCs or RECs.

### Mitochondrial donation assays

ρ^0^ A549 and ρ^0^ HeLa cell lines were labeled with 5 μL/mL of DiD (Invitrogen, USA) for 20 min at 37 °C. MSCs and RECs were labeled with 200 nM MitoTracker Green FM (Invitrogen, USA) for 20 min at 37 °C. Co-culture assays were done for 12 h in RPMI 1640 growth medium (Wako, Japan) supplemented with 1% penicillin–streptomycin and 10% fetal bovine serum (FBS; HyClone). Cells were then washed with phosphate-buffered saline (PBS) and detached by incubating cells in TrypLE™ Express Enzyme (Life technologies, Carlsbad, CA) for 5 min at 37 °C. Cellular viability was assessed using 4% trypan blue exclusion and light microscopy and was always greater than 95%. Specific dye transfer was measured by gating DiD events, excluding doublets (forward scatter width vs. height), and measuring fluorescence in the fluorescein channel. Cells were acquired using the CytoFLEX (BECKMAN COULTER, USA), and the data were analyzed using FlowJo™ Software Version 10 (BD, USA). Inhibitory compounds (vehicle control DMSO, 1 µL/mL; cytochalasin D, 350 nM; dynasore, 80 µM; Carbenoxelone, 100 µM) used to determine the mechanisms underlying mitochondrial transfer were added to culture media simultaneously with the MSCs throughout the duration of the assay.

### Fluorescence imaging

To examine the mitochondrial distribution, mitochondria in MSCs/RECs were stained using 200 nM MitoTracker Deep Red (Invitrogen, USA) for 20 min at 37 °C. ρ^0^ A549 and ρ^0^ HeLa cell nuclei was stained with 1 μg/mL DAPI (Invitrogen, USA) for 20 min at 26 °C. After washing three times with PBS, the cells were trypsinized and co-cultured in a 24-well plate for 24 h. Cells were then fixed with 4% paraformaldehyde (PFA) and permeabilized using 0.1% Triton X-100 in PBS. Next, 1X green fluorescent phalloidin conjugate working solution (Abcam, USA) was added to the fixed cells (100 μL/well, 96-well plate), and the cells were stained at room temperature for 60 min for microtubules/TNTs.

For immunofluorescence, the co-cultured cells were fixed with 4% PFA for 15 min and blocked in blocking buffer for 60 min. Cells were then incubated with primary anti-connexin43 (1:1000; Cell Signaling, USA)) in 1% BSA and 0.3% Triton X-100/PBS overnight at 4 °C. Rinsed twice in PBS for 15 min each incubated with Alexa-Fluor® 488 conjugated secondary antibodies (Invitrogen, USA) for 1 h at room temperature. After washed twice with PBS, they were mounted with ProLong Gold antifade reagent (Invitrogen, USA). Fluorescent staining was visualized under a BZ-X710 microscope (KEYENCE, Japan).

### Transmission electron microscopy (TEM)

Cells were grown in 35 mm dishes. After 10–12 h, cells were pre-fixed in 2.5% glutaraldehyde solution for electron microscopy (Wako, Japan), 2% PFA, and 0.1 M phosphate buffer (Wako, Japan) for 2 h at room temperature, washed three times with PBS, and dehydrated in an ascending gradual series of ethanol (50%, 70%, 90%, 95%, and 100%) for 8 min each. Cells were then mounted in an epoxy resin using propylene oxide to aid infiltration, which was hardened at 60 °C for 24 h. Ultrathin sections were taken using a diamond knife, mounted on copper mesh grids, stained with uranyl acetate, and imaged using a Topcon EB-002B transmission electron microscope.

### MtDNA content assay

Total DNA of the cultured cells was extracted using a QIAamp DNA Micro Kit (Qiagen, USA). mtDNA content was determined using polymerase chain reaction (PCR) and corrected by simultaneous measurements of nuclear DNA (nDNA), as described previously [27].

For reverse transcription-quantitative PCR (RT-qPCR), total RNA was extracted using ISOGEN-LS reagent (Nippon Gene, Japan) according to the manufacturer’s instructions. First-strand cDNA was synthesized from 2 μg of total RNA using PrimeScript II 1st Strand cDNA Synthesis Kit (TaKaRa, Japan). RT-qPCR was performed by the Eco™ Real-Time PCR System (Illumina, USA) and KAPATM SYBR FAST qPCR Mastermix (KAPA Biosystems Inc., USA). In the co-culture group, two different adherent cells were isolated using the transwell apparatus. The expression of NADH dehydrogenase 1 (ND1), cytochrome oxidase-1(COX-1), hypervariant, and region 2 (HVR2) was used to verify mtDNA loss. Used primers are listed in Table 1.

**Table 1:** Primer pairs to confirm establishment of ρ^0^ A549 and ρ^0^ HeLa cell lines

Gene name	Primer pairs	Product size (bp)
ND1 (mtDNA)	Forward 5′-ACC CCC GAT TCC GCT ACG ACC AAC-3′	164
	Reverse 5′-GGT TTG AGG GGG AAT GCT GGA GAT-3′	
COX1 (mtDNA)	Forward 5′-ACA CGA GCA TAT TTC ACC TCC G-3′′	336
	Reverse 5′-GGA TTT TGG CGT AGG TTT GGT C-3′	
HVR2 (mtDNA)	Forward 5′-CTC ACG GGA GCT CTC CAT GC-3’	401
	Reverse 5′-CTG TTA AAA GTG CAT ACC GCC A-3′	
CH1 (nDNA)	Forward 5′-GGC TCT GTG AGG GAT ATA AAG ACA-3′	98
	Reverse 5′-CAA ACC ACC CGA GCA ACT AAT CT-3′	
ACTB	Forward 5′-TGG CAC CCA GCA CAA TGA A-3′	186
	Reverse 5′-CTA AGT CAT AGT CCG CCT AGA AGC A-3′	

*mt* mitochondria, *n* nuclear

### Measurement of mitochondrial membrane potential (∆ψm)

Following the specific treatments, the ∆ψm of the different cell lines was assessed using a JC-1 MitoMP Detection Kit (Dojindo, Japan), exhibiting potential-dependent accumulation in the mitochondria. Briefly, cells (1 × 10^5^ cells/mL) were incubated with JC-1 for 45 min at 37 °C. Imaging buffer solution was added, and cells were observed under a fluorescence microscope. Fluorescence was detected using a GloMax® Discover Microplate Reader (Promega, USA). The excitation and emission wavelengths were 530 nm to detect the monomeric form of JC-1 and 590 nm to detect its aggregation. The ratio of mitochondrial JC-1 aggregates to monomers was considered representative of the ∆ψm of the cells.

### Measurement of intracellular ATP levels

ATP levels were determined using an Intracellular ATP assay kit ver.2 (Wako, Japan) according to the manufacturer's instructions. After 12 h of co-culture in the non-contact system, ρ^0^ cells (1 × 10^5^ cells) were washed twice with PBS and suspended in 100 μL of PBS. The cell suspension was inoculated into 96-well microtiter plates, followed by the addition of 100 μL of ATP assay reagent. After shaking for 1 min at room temperature, luminescence was measured using a GloMax® Discover Microplate Reader (Promega, USA).

### Measurement of intracellular lactate levels

Lactate levels were determined using a Lactate-GloTM Assay (Promega, USA), according to the manufacturer's instructions. After 12 h of co-culture, the supernatant of each group was collected and diluted 60 times. Next, 50 μL was transferred to 96-well assay plates, and 50 μL Lactate Detection reagent was added. The cells were incubated at room temperature for 60 min and luminescence was measured by the GloMax® Discover Microplate Reader (Promega, USA).

### Measurement of ROS levels

MtROS production was measured using the MitoSOX probe according to the manufacturer’s protocol (Life Technologies, USA). After 12 h of co-culture, the cells were seeded in a 24-well plate and incubated with MitoSOX Red (5 μM) for 10 min at 37 °C in the dark. After three gentle washes with 0.1% FBS in Hanks' balanced salt solution (HBSS), cells were cultured in HBSS and subjected to confocal imaging. Fluorescence intensity was measured by the GloMax® Discover Microplate Reader (Promega, USA).

### Analysis of cell proliferation

In the direct contact co-culture system, detached ρ^0^ A549 and ρ^0^ HeLa cells were labeled with 10 μM carboxyfluorescein-succinimidyl ester (CFSE) (Invitrogen, USA) at 37 °C for 20 min in the dark and washed twice with PBS containing 10% FBS to remove excess CFSE. Cells were plated at 1 × 10^5^ cells/well in 24-well plates and incubated at 37 °C with 5% CO_2_. At least 4 h after seeding, MSCs or RECs were added to each well at a density of 1 × 10^5^ cells/well, and the cells were further incubated for 72 h. At the appropriate time point, the cells were detached, washed twice, resuspended in buffer, and immediately analyzed using CytoFLEX (BECKMAN COULTER, USA).

In the non-contact co-culture system, the proliferation of ρ^0^ A549 and ρ^0^ HeLa cells after 0–96 h of treatment with MSCs or RECs was assessed using the cell counting kit-8 (CCK-8) assay. Briefly, cells were incubated with 2-(2-methoxy-4-nitrophenyl)-3-(4-nitrophenyl)-5-(2,4-disulfophenyl)-2H-tetrazolium (WST-8) (Dojindo, Japan) for 3 h in a humidified atmosphere (37 °C, 5% CO_2_). The absorbance of 450 nm was measured by the GloMax® Discover Microplate Reader (Promega, USA).

### Enzyme-linked immunosorbent assay (ELISA)

The cell culture media samples were prepared according to the manufacturer’s instructions and the levels of human growth/differentiation factor 15 (GDF-15) were detected using ELISA kit (Invitrogen, USA). The optical density of the samples was measured using a GloMax® Discover Microplate Reader at 450 nm.

### Measurement of oxygen consumption rate

The oxygen consumption rate (OCR) was examined using Seahorse XF Cell Mito Stress Test Kit and measured with the Seahorse XF HS mini (Seahorse Bioscience, Agilent, USA) according to the manufacturer instructions. Briefly, cells were evenly seeded (1.5 × 10^4^ cells/well) onto the XF HS mini cell culture plate. Cells were equilibrated in unbuffered RPMI medium (Seahorse Bioscience, Agilent, USA) supplemented with 10 mM glucose, 1 mM sodium pyruvate, and 2 mM l-glutamine, and transferred to a non-CO_2_ incubator for 1 h. Coupled and uncoupled respiration were first measured using 1.5 μM oligomycin (Olig). Maximal respiration capacity was determined using 1 μM FCCP. Finally, non-mitochondrial respiration was determined using a combination of 0.5 μM antimycin A and Rotenone (AA/Rot). Each plotted value of the real-time assessment of mitochondrial respiration was represented as the percentage of basal OCR. Datasets were analyzed using Wave software (Agilent, USA).

### Statistical analysis

Data analysis was performed using the GraphPad Prism software version 9.0 (San Diego, CA). Data are expressed as the mean ± standard deviation. Student’s t-test or one-way ANOVA with Tukey’s post hoc analysis was performed to compare the differences between two or more groups, and *p* < 0.05 was considered to be statistically significant.

## Results

### Mitochondrial transfer efficiency from MSCs/RECs to ρ^0^ cells in the direct contact system

**Fig. 2:**
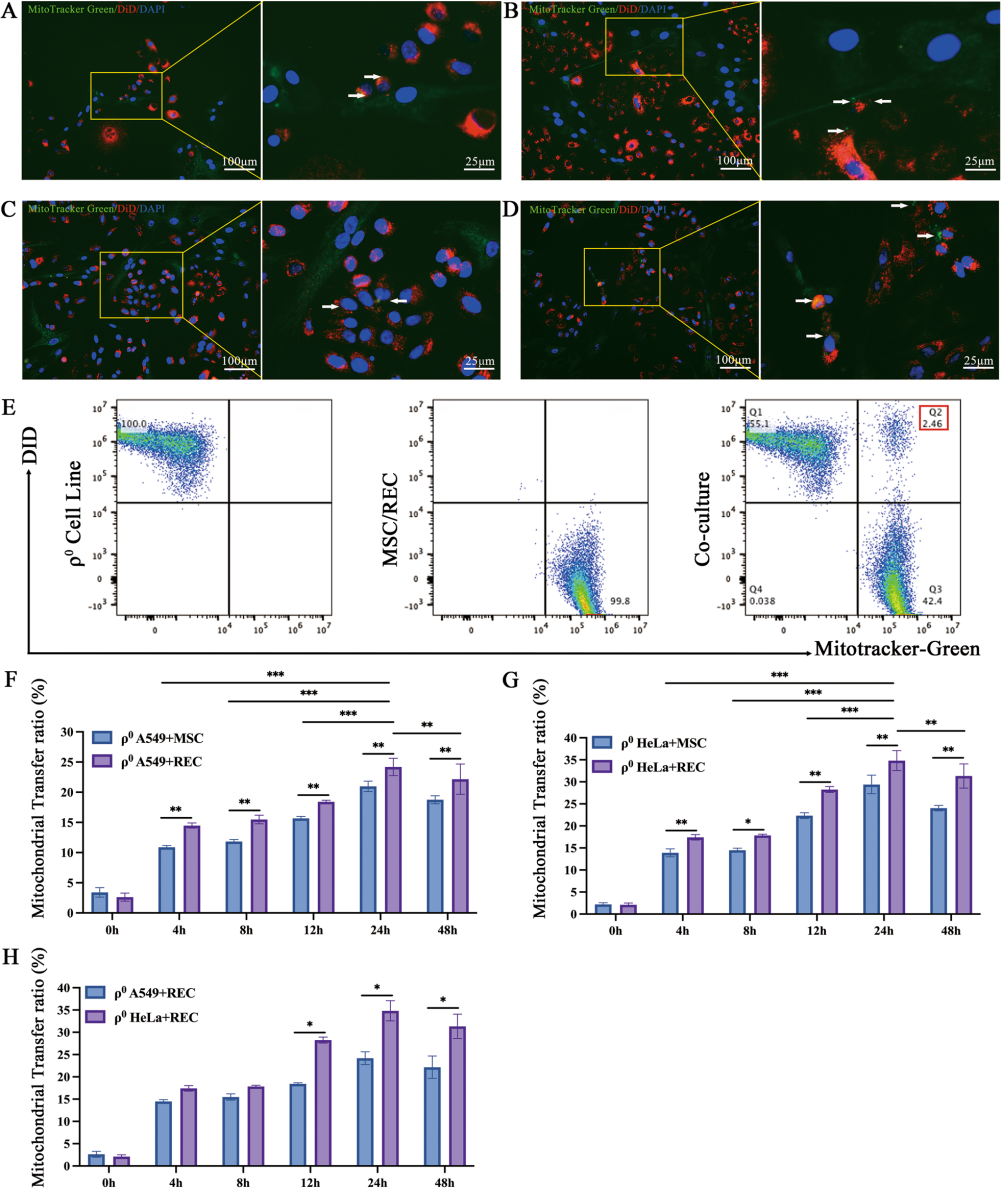
Mitochondrial transfer from MSCs/RECs to ρ^0^ cells. **A** Representative image of co-culture showing ρ^0^ A549 cells (red) with MSC (green). **B** Representative image of co-culture showing ρ^0^ A549 cells (red) with the REC (green). **C** Representative image of co-culture showing ρ^0^ HeLa cells (red) with the MSC (green). **D** Representative image of co-culture showing ρ^0^ HeLa cells (red) with the REC (green). Arrowhead: green fluorescence of mitochondria from MSC/REC transmitted to the ρ^0^ cells containing red fluorescence, indicating successful transference. **E** Representative distribution of DiD-labeled ρ^0^ cells (red) and MitoTracker-labeled MSC/REC (green) in co-cultured cells analyzed by FCM. **F** The time course of the mitochondrial transfer ratio between REC/MSC and ρ^0^ A549 cells distributed at the Q2 phase and the percentage of this population over the total ρ^0^ A549 cells are presented. **G** The time course of the mitochondrial transfer ratio between REC/MSC and ρ^0^ HeLa cells distributed at the Q2 phase and the percentage of this population over the total ρ^0^ HeLa cells are presented. **H** Differences in the rate of mitochondrial transfer by REC to different ρ^0^ cell lines between 0 and 48 h are shown. Data were based on three independent experiments. *p < 0.05, **p < 0.01, ***p < 0.001

To examine whether RECs have the ability to transfer of own mitochondria to the mtDNA- deficient ρ^0^ cells. DiD-labeled ρ^0^ cells (red) and MitoTracker-labeled MSCs/RECs (green) were co-culture for 24 h. Several ρ^0^ cells exhibiting red fluorescence also contained green fluorescence mitochondrial structures (Fig. 2A–D). In addition, some ρ^0^ cells received multiple clusters of mitochondria were also observed (Fig. 2A, D). FCM analysis showed that DiD/MitoTracker double positive population was visible by co-culture (Fig. 2E). Further, the ratio of RECs transferred to mitochondria was significantly increased until 24 h in both ρ^0^ A549 and ρ^0^ Hela cells, and those were significantly higher in REC than conventional BM-MSCs (Fig. 2F, G). At the same time, we compared the effect of RECs on mitochondrial transfer rates of two different ρ^0^ cell lines, and found that ρ^0^ HeLa cells received more mitochondria donated by REC (Fig. 2H). These data indicated that the RECs was capable of more actively transferring mitochondria to ρ^0^ cells compared with conventional BM-MSCs.

### MSCs/RECs restored the morphology of mtDNA-deficient ρ^0^ cells in contact system

**Fig. 3:**
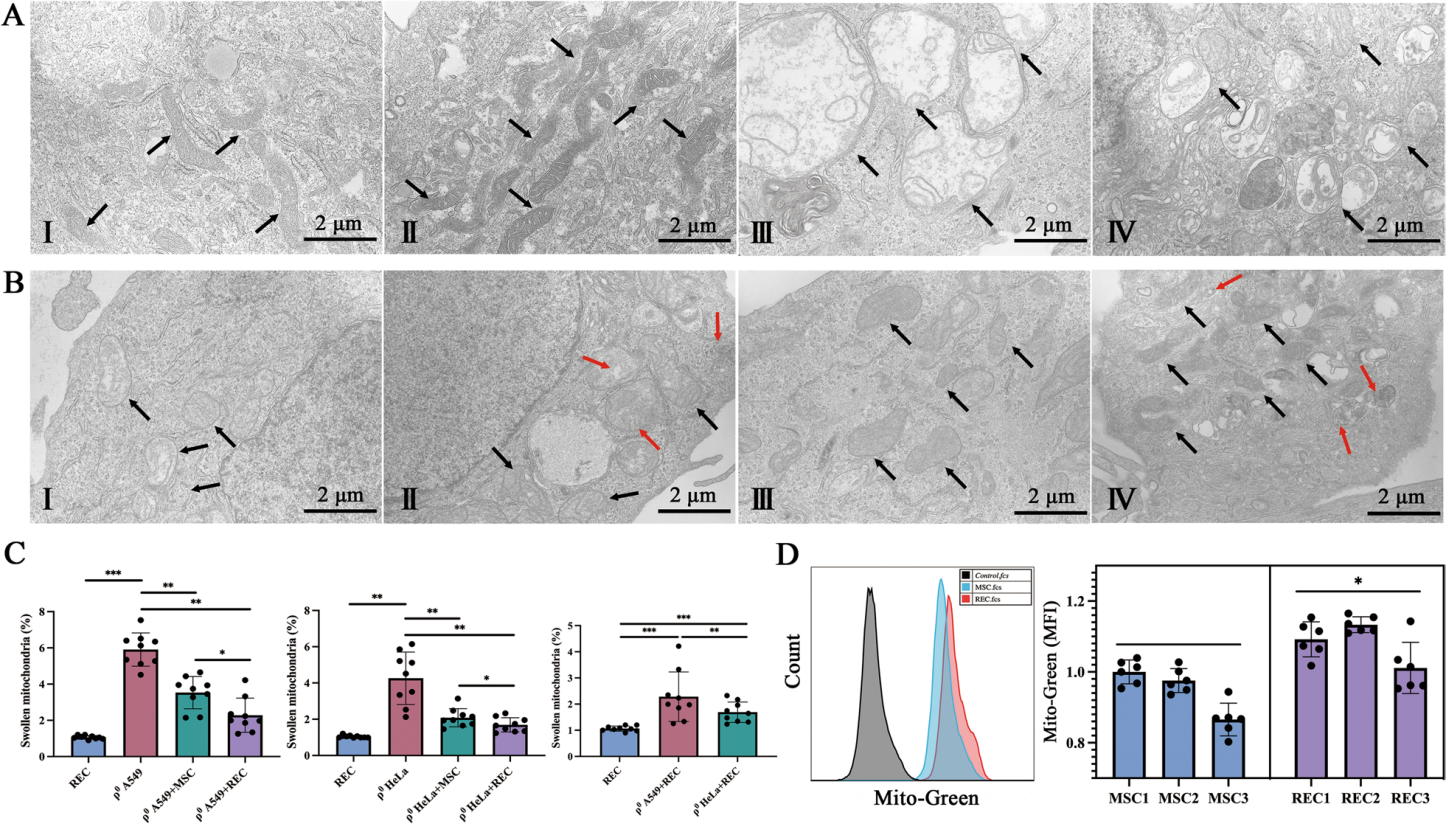
Transmission electron microscopic image of mitochondria in the direct contact system. **A** (**I–IV**) Represent mitochondria in MSC, REC, ρ^0^ A549, and ρ^0^ HeLa, respectively. **B** Shows the mitochondrial morphology of ρ^0^ A549 cells after 24 h co-culture with MSC (I) and REC (**II**). **B** Shows the mitochondrial morphology of ρ^0^ HeLa cells after 24 h co-culture with MSC (**III**) and REC (**IV**). The black arrow points to mitochondria. Red arrows point to mitochondria in microvesicles. This was confirmed in three independent ρ^0^ cells. **C** Quantification of swollen mitochondria. **D** Flow cytometry quantification of mitochondria from MitoTracker Green-labeled MSC and REC following their 24-h incubation. Data were based on three independent experiments. *p < 0.05, **p < 0.01, ***p < 0.001

In the contact co-culture system, the morphology and intracellular distribution of mitochondria in each group of cells were observed by TEM (representative images in Fig. 3A, B). A large number of normal mitochondria were found in MSCs and RECs, with uniform spherical or rod-like morphology (Fig. 3A, I–II). The number of swollen mitochondria in ρ^0^ cells was found to be significantly higher than that in the REC group (Fig. 3C). Additionally, swollen mitochondria displayed disordered cristae, and interdigitation was disrupted (Fig. 3A, III–IV; black arrows). These observations support the hypothesis that mitochondrial health is affected by mtDNA depletion. After co-culture with MSCs or RECs, the mitochondrial morphology of ρ^0^ cells returned to normal significantly, and the mitochondrial swelling of ρ^0^ cells in the co-culture group with RECs was significantly reduced (Fig. 3B, I–IV and 3C). Mitochondria were found in microvesicles (Fig. 3B, II, IV; red arrow) in ρ^0^ cells co-cultured with RECs, but no mitochondria in microvesicles were found in ρ^0^ cells co-cultured with MSCs. Furthermore, we observed that RECs had more mitochondria than MSCs (Fig. 3A, I–II), so we assessed the mean fluorescence intensity of Mito-Green in MSCs and RECs under the same conditions. RECs showed a higher Mito-Green fluorescence intensity (Fig. 3D). We further verified that RECs had higher mitochondrial content.

### Mitochondrial transfer pathways of MSCs and RECs in contact systems

**Fig. 4:**
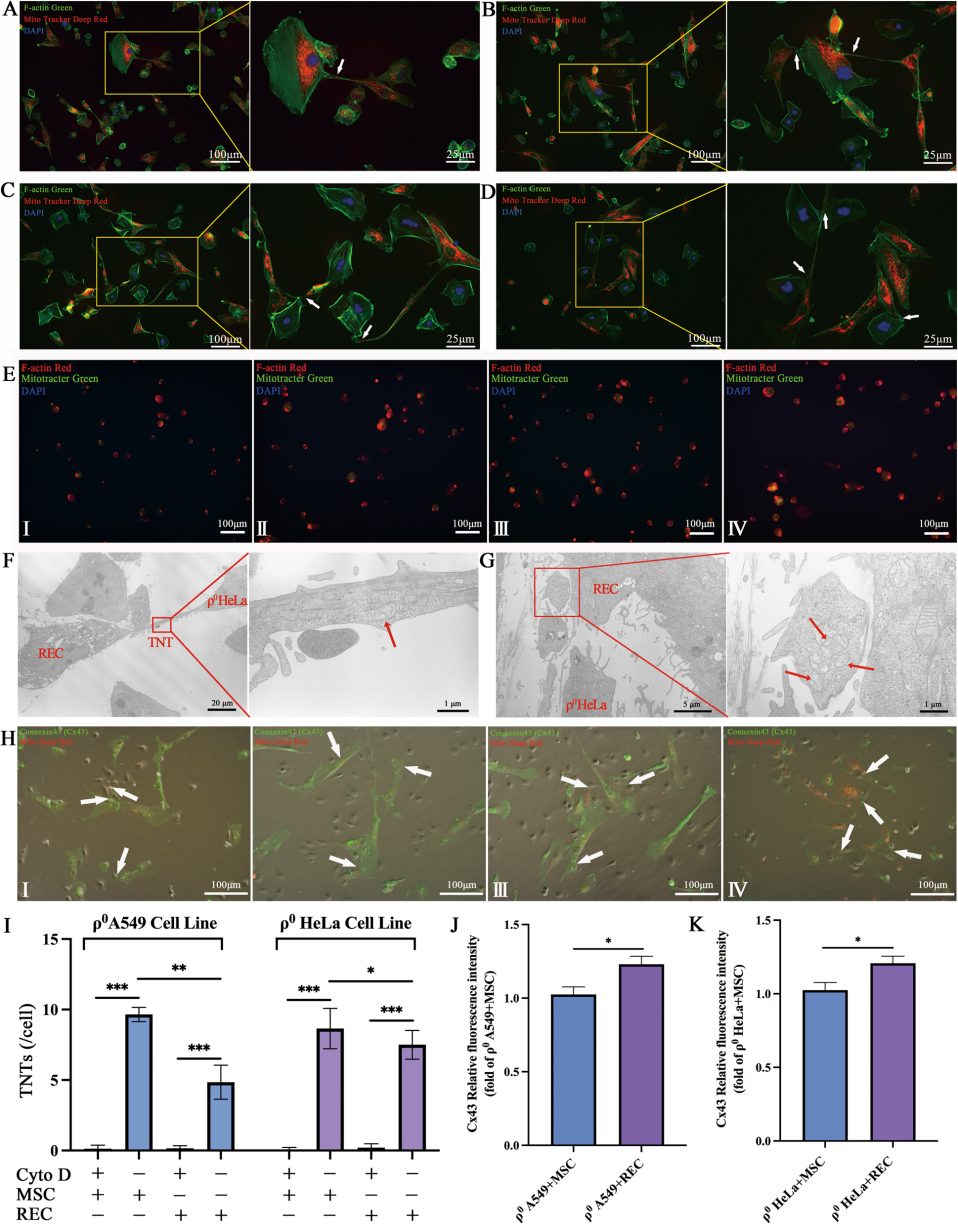
MSC/REC connect with ρ^0^ cells through F-actin containing microtubules and gap junction. Representative immunofluorescent images of (**A**) ρ^0^ A549 co-culture with MSC, (**B**) ρ^0^ A549 co-culture with REC, (**C**) ρ^0^ HeLa co-culture with MSC, and (**D**) ρ^0^ HeLa co-culture with REC. MSC/REC (red) were labeled with Mito Tracker Deep Red, ρ^0^ cells were labeled with DAPI (blue), and all cells were counterstained with phalloidin (F-actin, green). White arrows depict microtubules connecting MSC/REC and ρ^0^ cells. **E** Cytochalasin D was an inhibitor of actin formation, added during ρ^0^ A549 co-culture with MSC (I) and REC (**II**), respectively. Cytochalasin D was added during ρ^0^ HeLa co-culture with MSC (**III**) and REC (**IV**), respectively. MSC/REC (green) were labeled with Mito Tracker Green, ρ^0^ cells were labeled with DAPI (blue), and all cells were counterstained with phalloidin (F-actin, red). **F** Mitochondria in TNTs were observed by transmission electron microscopy. **G** Mitochondria in microvesicles were observed by transmission electron microscopy. Representative immunofluorescent images of (**H**) ρ^0^ A549 co-culture with MSC (I) and REC (**II**), respectively. (**III–IV**) ρ^0^ HeLa co-culture with MSC (**III**) and REC (**IV**), respectively. MSC/REC (red) were labeled with Mito Tracker Deep Red and all cells were counterstained with connexin 43 (Gap junction, green). The white arrow shows MSC/REC generating gap junction to transfer mitochondria. **I** When exposed to Cytochalasin D, the number of TNTs in the ρ^0^ A549 cell line and ρ^0^ HeLa cell line was calculated. **J** Quantitative analysis of the relative fluorescence intensity of Cx43 protein produced by ρ^0^ A549 cells co-cultured with MSC and REC, respectively. **K** Quantitative analysis of the relative fluorescence intensity of Cx43 protein produced by ρ^0^ HeLa cells co-cultured with MSC and REC, respectively. Data were based on three independent experiments. *p < 0.05, **p < 0.01, ***p < 0.001

Previous reports demonstrated that the F-actin-modulated formation of TNTs contributes to mitochondrial transfer from BM-MSCs to macrophage and improves macrophage mitochondrial respiration [28]. We examined the invelvement of TNTs in the regulation of MSCs and RECs mitochondrial transfer in vitro. MitoTracker Deep red-labeled MSCs/RECs (red) were co-cultured with DAPI-labeled ρ^0^ cells. After 24 h, staining with phalloidin, a high-affinity F-actin probe, showed that ρ^0^ cells and MSCs or RECs were bridged by TNTs, which allowed the effective transfer of MSCs or RECs mitochondria to injured ρ^0^ cells (Fig. 4A–D), thereby suggesting that TNTs are vital for mitochondrial transfer. These projections stained strongly with phalloidin, indicating that they comprised F-actin, consistent with previous reports on MSCs [29]. Electron microscopy results also showed that RECs donated healthy mitochondria to ρ^0^ cells through TNTs (Fig. 4F). In addition, the transfer of mitochondria to ρ^0^ HeLa cells from RECs in microvesicles under endocytosis was also observed using TEM and mitochondrial dynamics (Fig. 4G, Additional file 1: Video S1). To determine whether ρ^0^ cells could take up leaked mitochondria from MSCs without cell–cell connection, we treated cells with cytochalasin D (Fig. 4E), which causes F-actin aggregation and retards TNTs formation by inhibiting actin polymerization and philopodia elongation without affecting endocytosis [19, 30]. Administration of cytochalasin D (350 nM) did not affect MSC viability, but led to no TNTs formation by MSCs/RECs (Fig. 4E, I). Additionally, TNTs formation was significantly lower in RECs than that in MSCs (Fig. 4I). Further, we identified the role of connexin-43 in mitochondrial transfer where we examined the transfer of mitochondria to ρ^0^ cells by gap junctions generated by MSCs or RECs (Fig. 4H). Connexin-43 was almost not expressed in ρ^0^ cells, consistent with a previous report [31]. The relative fluorescence intensity of Cx43 was quantified in each group, and it was found that the relative fluorescence intensity of Cx43 was significantly increased after RECs was co-cultured with ρ^0^ cells (Fig. 4J–K).

### Effects of inhibitors on mitochondrial transfer

**Fig. 5:**
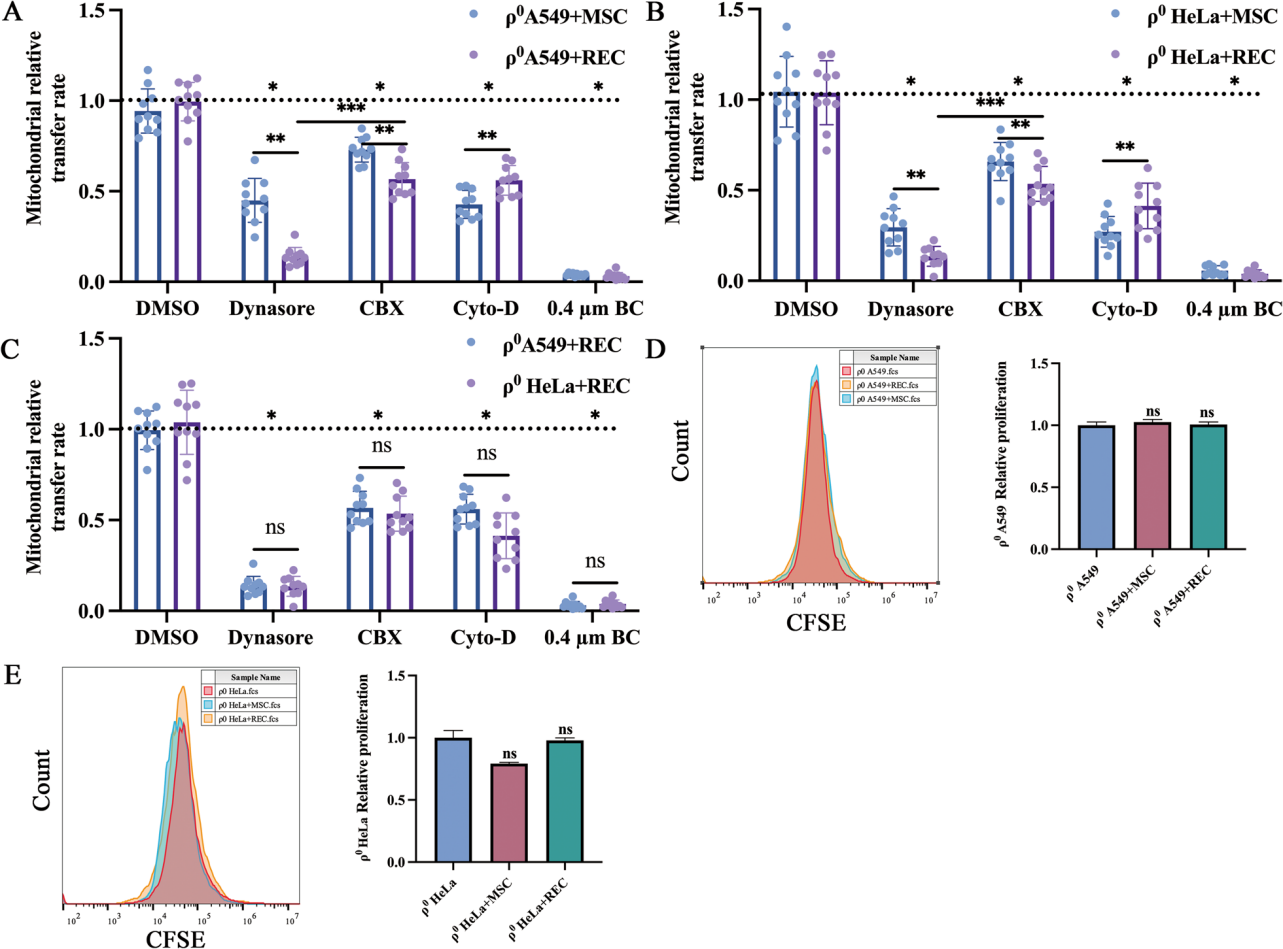
Mitochondrial transfer rates of REC and MSC to ρ^0^ cells under various inhibitors. MSC and REC donate the mitochondria to ρ^0^ A549 cells (**A**) and ρ^0^ HeLa cells (**B**) via multiple mechanisms. MSC and REC were co-cultured with ρ^0^ cells and treated with either Dynasore to inhibit endocytosis, Cytochalasin D (Cyto-D) to inhibitor TNT formation, Carbenoxolone (CBX) to inhibit all gap junction formation, and 0.4 µm Boyden chamber (0.4 µm BC) inhibits direct cell-to-cell contact. **C** REC donate the mitochondria to ρ^0^ A549 cells and ρ^0^ HeLa cells via multiple mechanisms. A horizontal line represents uninhibited control. The proliferation of ρ^0^ A549 cells (**D**) and ρ^0^ HeLa cells (**E**) when co-cultured with MSC or REC. carboxyfluorescein-succinimidyl ester (CFSE) labels ρ^0^ cells. Data were based on three independent experiments. *p < 0.05, **p < 0.01, ***p < 0.001. ns: no statistical significance between the indicated groups

Inhibiting endocytosis in ρ^0^ A549 cells resulted in a 49.3% and 85.5% decrease in mitochondrial transfer in MSC and REC co-cultures, respectively (Fig. 5A, B). In ρ^0^ HeLa cells, 74.8% and 90.4% decreases were found in MSCs and RECs co-cultures, respectively (Fig. 5A, B). Inhibiting microtubules/TNTs formation reduced mitochondrial transfer by 51.5% and 43.5% in ρ^0^ A549 cells co-cultured with MSCs and RECs, respectively (Fig. 5A, B). 77.3% and 62.5% reduction was identified in ρ^0^ HeLa cells co-cultured with MSCs and RECs, respectively (Fig. 5A, B). Gap junction inhibition decreased mitochondrial donation by 19.4% and 42.8% in ρ^0^ A549 cells co-cultured with MSCs and RECs, respectively (Fig. 5A, B). In ρ^0^ HeLa cells co-cultured with MSCs and RECs, gap junction inhibition decreased mitochondrial donation by 38.5% and 53.4% (Fig. 5A, B). The mitochondrial transfer rate of RECs cocultured with ρ^0^ A549 cells or ρ^0^ HeLa cells after endocytosis inhibitor was significantly lower than that of gap junction inhibitor. When we used a 0.4 μm Boyden chamber to inhibit all mitochondrial transfer pathways, we observed more significant attenuation of mitochondrial transfer (Fig. 5A, B), suggesting synergy between microtubules/TNTs, endocytosis, and gap junction-mediated mechanisms. RECs may be more sensitive to endocytosis inhibitors. Regarding the transfer rate of different types of ρ^0^ mitochondria by RECs, we found no significant difference in the rate of mitochondrial donation by RECs to the two types of ρ^0^ cells under each group of inhibitors (Fig. 5C). However, no effect was observed on the proliferation of ρ^0^ cells after co-culture with MSCs or RECs (Fig. 5D, E).

### MSCs/RECs restored the morphology of mtDNA-deficient ρ^0^ cells in the Non-contact co-culture system

**Fig. 6:**
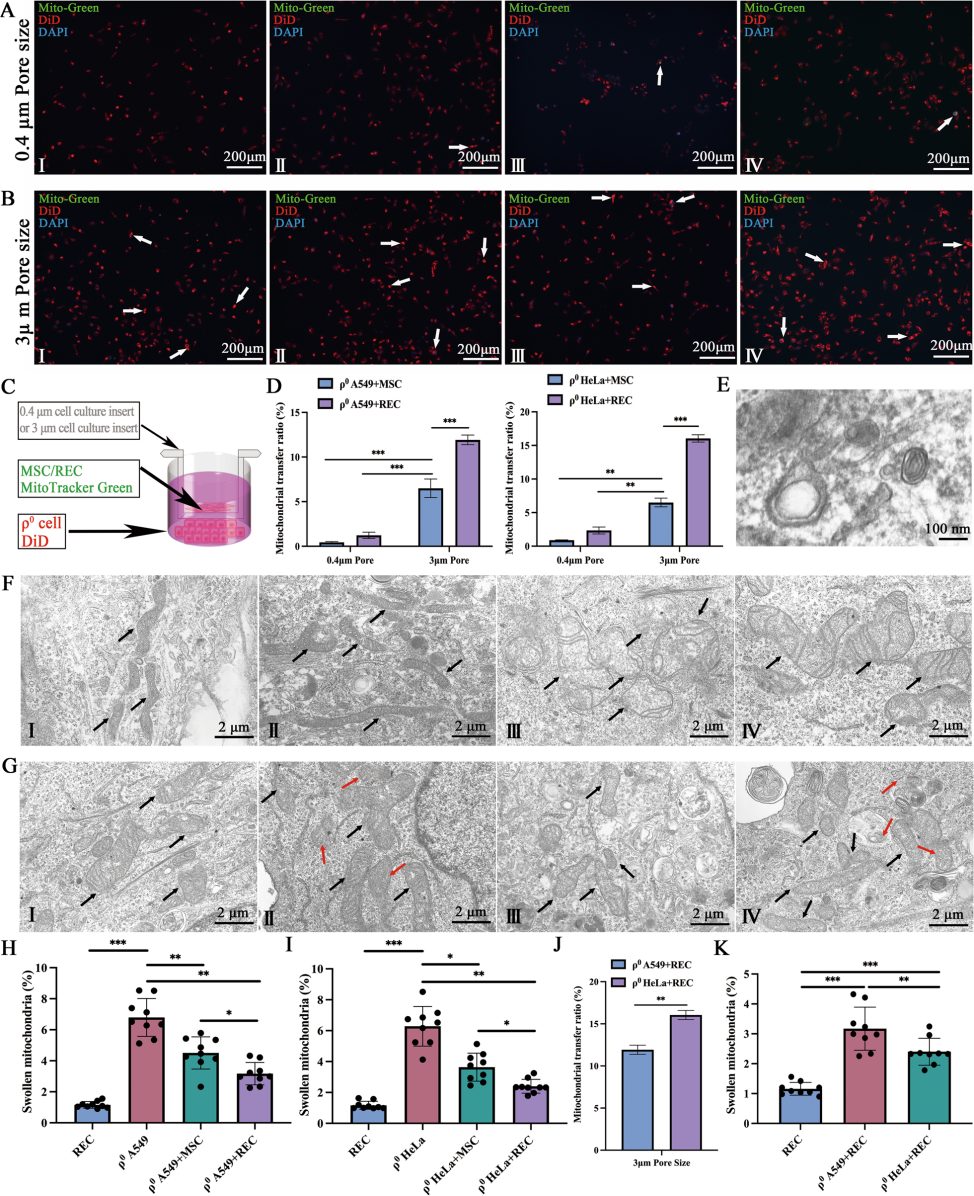
Mitochondrial transfer and mitochondrial morphology in non-contact co-culture system. Using the culture system as shown in (**C**), MSC or REC were seeded in cell culture insert, and ρ^0^ cells were seeded in the lower petri dish. **A** In 0.4 μm pore size cell culture insert, ρ^0^ A549 co-culture with MSC (I**)** and REC (**II**), respectively. ρ^0^ HeLa co-culture with MSC (**III)** and REC (**IV**), respectively. **B** In 3 μm pore size cell culture insert, ρ^0^ A549 co-culture with MSC (I**)** and REC (**II**), respectively. ρ^0^ HeLa co-culture with MSC (**III)** and REC (**IV**), respectively. MSC/REC (green) were labeled with Mito Tracker Green and ρ^0^ cells were labeled with DiD (red). In 0.4 μm pore size cell culture insert and 3 μm pore size cell culture insert, the mitochondrial transfer rate of ρ^0^ A549 cells and ρ^0^ HeLa cells (**D**) after 24 h co-culture with MSC and REC, respectively. **E** Representative TEM micrographs of exosomes from REC. **F** Represent mitochondria of MSC (I**)**, REC (**II**), ρ^0^ A549 (**III)**, and ρ^0^ HeLa (**IV**), respectively. **G** shows the mitochondrial morphology of ρ^0^ A549 cells after 24 h co-culture with MSC (I) and REC (**II**) and that shows the mitochondrial morphology of ρ^0^ HeLa cells after 24 h co-culture with MSC (**III**) and REC (**IV**). The black arrow points to the mitochondria. Red arrows point to mitochondria in microvesicles. Quantification of swollen mitochondria in ρ^0^ A549 cells (**H**) and ρ^0^ HeLa cells (**I**). **J** Quantification of swollen mitochondria in different types of ρ^0^ cell lines by co-culture with REC. **k** In 3 μm pore size cell culture insert, the mitochondrial transfer rate of ρ^0^ A549 and ρ^0^ HeLa cells after 24 h co-culture with REC. Data were based on three independent experiments. *p < 0.05, **p < 0.01, ***p < 0.001

In the non-contact co-culture system, we selected two different sizes of cell culture inserts (Fig. 6C). The 0.4 μm cell culture insert almost blocked most of the mitochondrial transfer (Fig. 6A), while the 3 μm cell culture insert allowed the passage of mitochondria, microvesicles, and exosomes (Fig. 6B). We found that the transfer of mitochondria to ρ^0^ cells in a 3 μm cell culture insert more in RECs (Fig. 6D). The exosomes with typical double-layered membrane vesicles of RECs were observed under TEM (Fig. 6E). Meanwhile, we also observed that the number of swollen mitochondria in ρ^0^ cells was significantly higher than that in the MSCs and RECs groups (Fig. 6F, I–IV). These swollen mitochondria had irregular cristae and interdigitation was disturbed (Fig. 6F, III–IV). After co-culture with MSCs or RECs for 24 h in a non-contact co-culture system, the mitochondrial morphology of ρ^0^ cells significantly returned to normal, and the mitochondrial swelling of ρ^0^ cells in the co-culture group with RECs was significantly reduced (Fig. 6G, I–IV). In addition, mitochondria in microvesicles were identified in ρ^0^ cells co-cultured with RECs (Fig. 6G, II and IV; red arrow), but essentially no mitochondria in microvesicles were found in ρ^0^ cells co-cultured with MSCs. We also examined the difference in the mitochondrial transfer rate of different types of ρ^0^ cells by RECs. We found that RECs also donated more mitochondria to ρ^0^ HeLa cells in the non-contact co-culture system (Fig. 6J), and ρ^0^ HeLa cells had fewer swollen mitochondria than ρ^0^ A549 cells after co-culture with RECs (Fig. 6K).

### MtDNA replenishment in ρ^0^ cells in the non-contact co-culture system

**Fig. 7:**
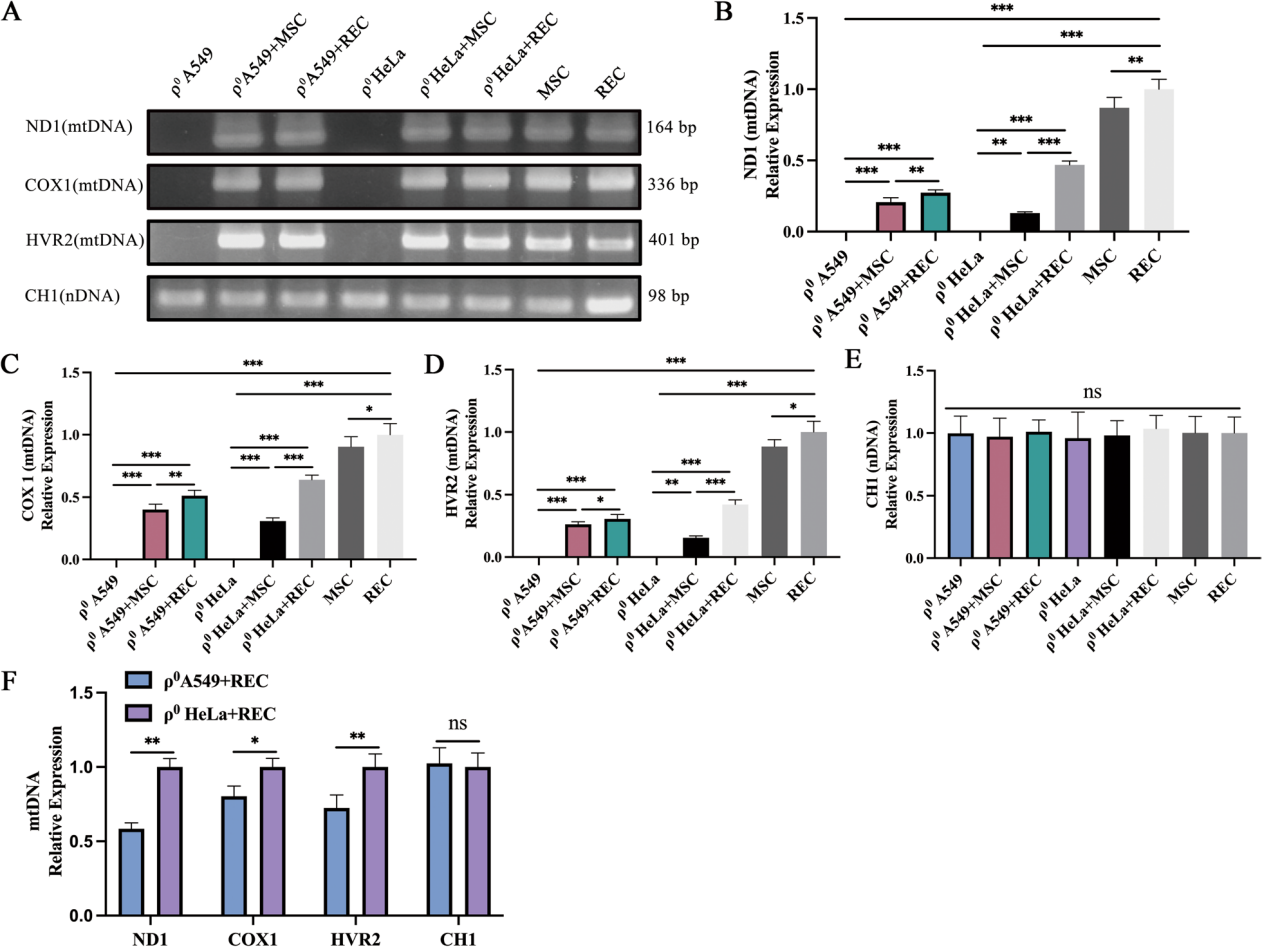
MtDNA recovery of ρ^0^ cells in the non-contact co-culture system. ρ^0^ cells acquired MSC and REC-derived mtDNA. **A** Total DNA from ρ^0^ cells, ρ^0^ + MSC cells, ρ^0^ + REC cells, REC, and MSC were isolated and analyzed by PCR for the presence of NADH dehydrogenase 1 (ND1), Cytochrome c oxidase I (COX1), and hypervariant region 2 (HVR2) in mtDNA and chromosome 1 segment for nuclear DNA (nDNA). Full-length gels are presented in Additional file 2. Note that the ρ^0^ cells exhibited no detectable mtDNA. Isolated RNA from ρ^0^ cells, ρ^0^ + MSC cells, ρ^0^ + REC cells, REC, and MSC were quantitatively analyzed using RT-qPCR for ND1 (**B**), COX1 (**C**), HVR2 (**D**) mtDNA content, and CH1 (**E**) nuclear DNA content. **F** RT-qPCR quantitatively analyzes the effect of REC on mtDNA and nDNA content in different ρ^0^ cell lines. All co-culture groups were in the non-contact co-culture system. Data were based on three independent experiments. *p < 0.05, **p < 0.01, ***p < 0.001

The establishment of ρ^0^ A549 and ρ^0^ HeLa cell lines was confirmed by PCR (Fig. 7A) using gene-specific primers (Table 1). Further, genetic analysis revealed that the mtDNA ND1, COX 1, and HVR2 content of ρ^0^ cells were restored after co-culture with MSCs and RECs (Fig. 7A). REC replenished more mtDNA to ρ^0^ cells than MSCs (Fig. 7B–D). There was no significant difference in the nDNA content among the groups (Fig. 7E). This is consistent with a previous report [32], indicating that RECs is more advantageous in restoring mtDNA of mitochondria-deficient cells. After RECs was co-cultured with ρ^0^ A549 and ρ^0^ HeLa cells, the mtDNA content of ρ^0^ HeLa cells was significantly restored by RECs (Fig. 7F).

### Mitochondrial transfer of MSCs and RECs can restore mitochondrial function of ρ^0^ cells partially

**Fig. 8:**
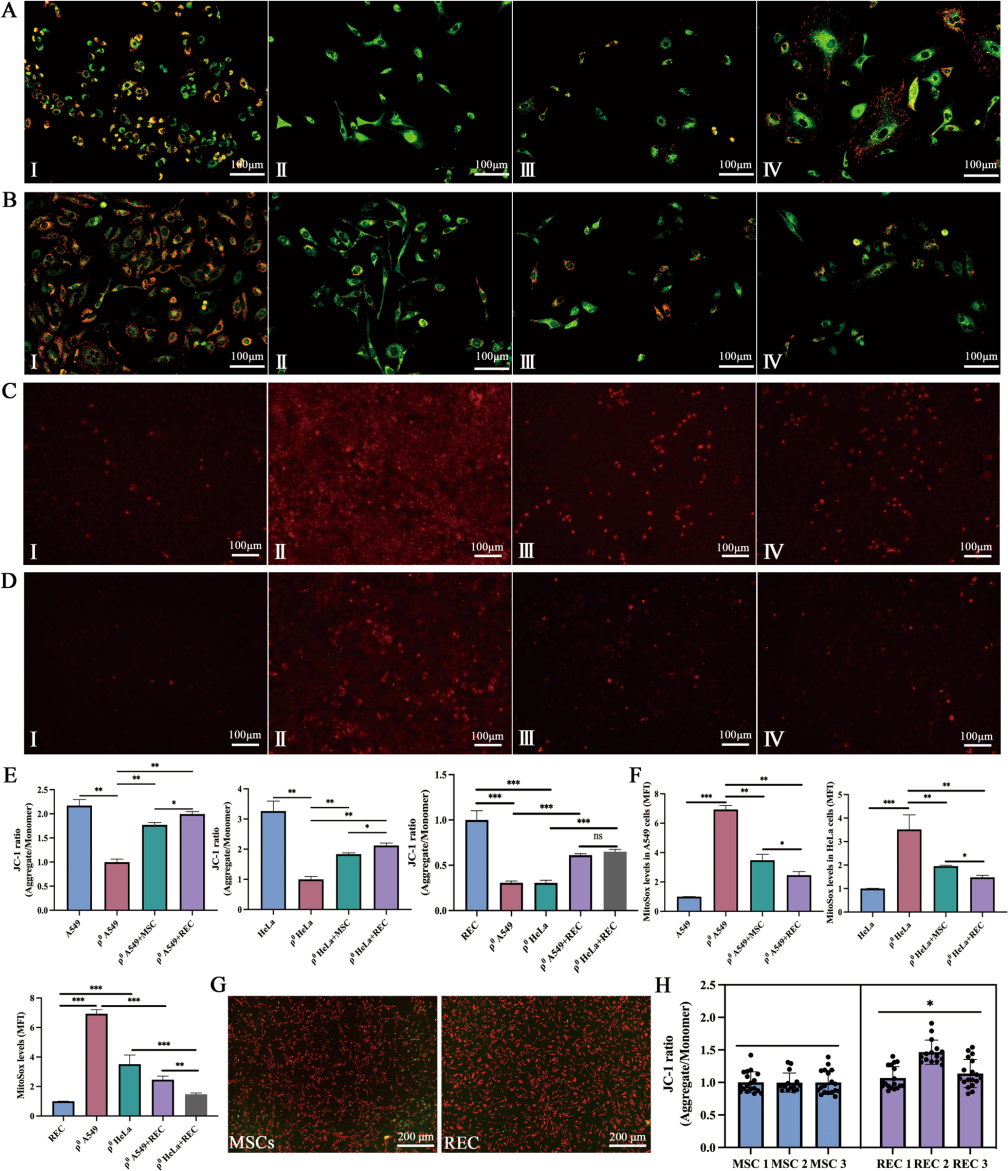
Mitochondrial function recovery of ρ^0^ cells after 24 h co-culture in non-contact system. **A** Fluorescence imaging of mitochondrial membrane potential in (I**–IV**) A549, ρ^0^ A549, ρ^0^ A549 + MSC, and ρ^0^ A549 + REC groups. **B** Fluorescence imaging of mitochondrial membrane potential in (I**–IV**) HeLa, ρ^0^ HeLa, ρ^0^ HeLa + MSC, and ρ^0^ HeLa + REC groups. **C** Representative images of Mito-Sox staining in (I**–IV**) A549, ρ^0^ A549, ρ^0^ A549 + MSC, and ρ^0^ A549 + REC groups. **D** Representative images of Mito-Sox staining in (**I–IV**) HeLa, ρ^0^ HeLa, ρ^0^ HeLa + MSC, and ρ^0^ HeLa + REC groups. **E** The flluorescence intensity ratio of mitochondrial membrane potential levels (JC-1) in different groups. **F** Quantitative analysis of ROS generation in different groups. **G** Representative fluorescence imaging of mitochondrial membrane potential in MSCs and RECs. **H** The fluorescence intensity ratio of mitochondrial membrane potential levels in MSCs and RECs. All co-culture groups were in a non-contact co-culture system. Data were based on three independent experiments. *p < 0.05, **p < 0.01, ***p < 0.001

The mitochondrial function of ρ^0^ cells was detected using a lipophilic and cationic dye—JC-1, and the results were expressed as a ratio of red (aggregated JC-1) and green (monometric JC-1) fluorescence (Fig. 8A, B). Quantitative analysis showed that ∆ψm increased significantly during co-culture treatment with MSCs and RECs compared to that in ρ^0^ cells alone. Following co-culture with MSCs and RECs, RECs exhibited a significantly higher ∆ψm than that by MSCs (Fig. 8E). In addition, RECs had a higher mitochondrial membrane potential than MSCs (Fig. 8G, H). The ROS-reactive fluorescence probe showed a significant decrease in the intracellular ROS levels after co-culture with MSCs or RECs (Fig. 8C, D), and the ROS levels in ρ^0^ cells co-cultured with RECs were significantly decreased compared to that in ρ^0^ cells co-cultured with MSCs (Fig. 8F). In ρ^0^ cells co-cultured with RECs, the intracellular ROS level of ρ^0^ HeLa was significantly lower than that of ρ^0^ A549 cells (Fig. 8F).

### ρ^0^ cells that received mtDNA from MSCs or RECs exhibited increased respiratory capacity

**Fig. 9:**
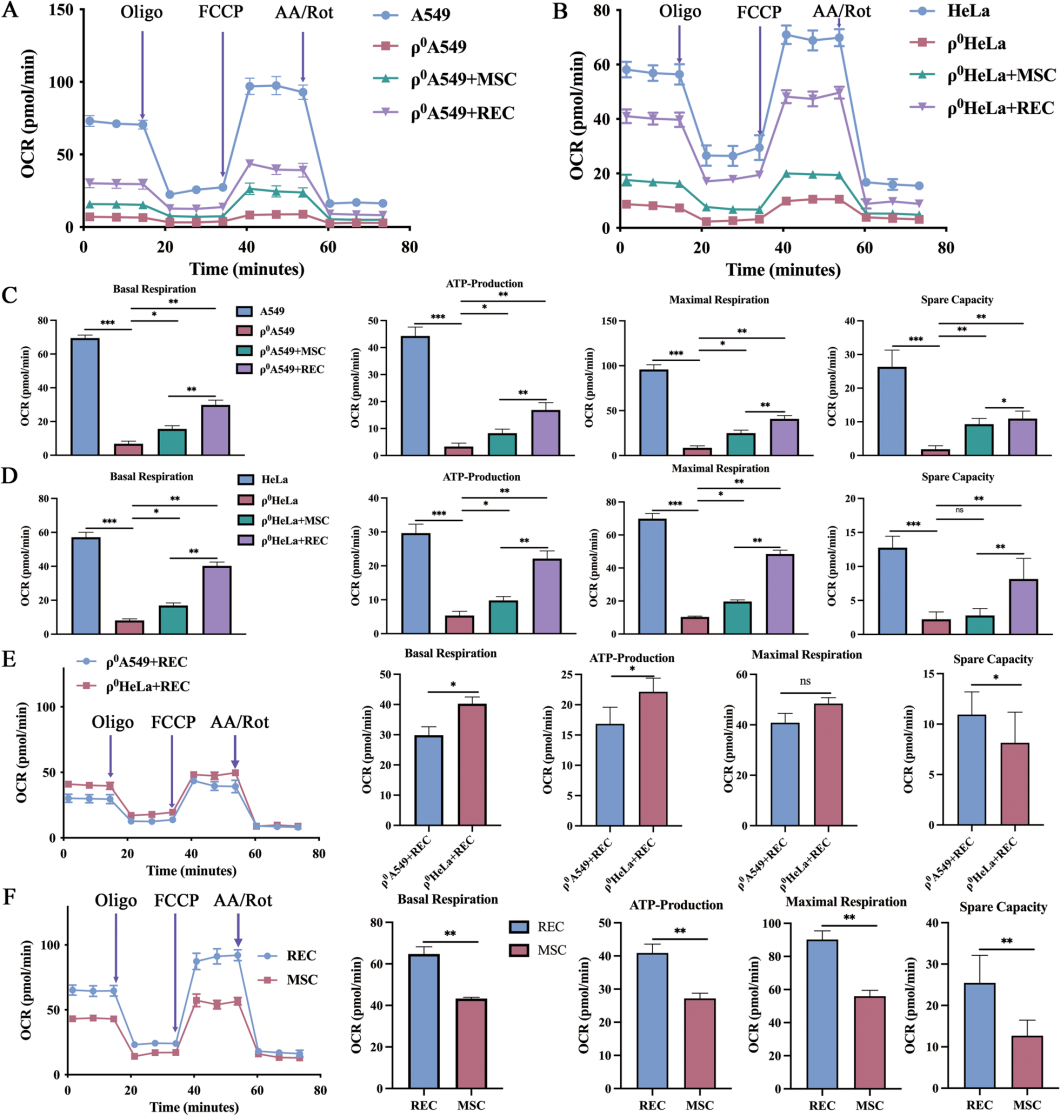
Recovery of cellular respiration activity in ρ^0^ cells that received mtDNA from MSCs/RECs. Oxygen Consumption Rate (OCR) of A549 (**A**) and HeLa (**B**) cell lines from different groups was measured over time (minutes). The injection order of Oligomycin (Olig), FCCP, and antimycin A and Rotenone (AA/Rot) are shown. Basal mitochondrial OCR, ATP production, and maximum respiration of A549 (**C**) and HeLa (**D**) cell lines from different groups were calculated. **E** OCR of ρ^0^ A549 + REC and ρ^0^ HeLa + REC groups was measured over time (minutes). Basal mitochondrial OCR, ATP production, and maximum respiration of MSC and REC were calculated. **F** OCR of MSC and REC groups was measured over time (minutes). Basal mitochondrial OCR, ATP production, and maximum respiration of MSC and REC were calculated. All co-culture groups were in non-contact co-culture system. Data were based on three independent experiments. *p < 0.05, **p < 0.01, ***p < 0.001

Given that mitochondria are a major contributor to cellular bioenergetics and ATP production via oxidative phosphorylation, we determined the respiratory function of the transferred mitochondria and the OCR of each group of cells. The bioenergetic profiles of the different groups are shown in Fig. 9A, B. Compared with control cells, basal mitochondrial OCR, ATP production, spare capacity, and maximum respiration were significantly decreased in ρ^0^ cells; however, these parameters recovered significantly in ρ^0^ cells after co-culture with MSCs or RECs (Fig. 9C, D), wherein ρ^0^ cells co-cultured with RECs showed a substantial improvement compared with those co-cultured with MSCs (Fig. 9C, D). And among the two kinds of ρ^0^ cells co-cultured with RECs, we found that the measured oxygen consumption rate of ρ^0^ HeLa cells showed that the production of basic mitochondrial OCR, ATP production, and spare capacity was significantly higher than those of the ρ^0^ A549 cells (Fig. 9E). Moreover, the oxygen consumption rates measured in MSCs and RECs showed a significantly higher basal mitochondrial OCR, ATP production, spare capacity, and maximum mitochondrial respiratory function in RECs as compared to that in MSCs (Fig. 9F).

### RECs reduced the GDF-15 level in the ρ^0^ cells

**Fig. 10:**
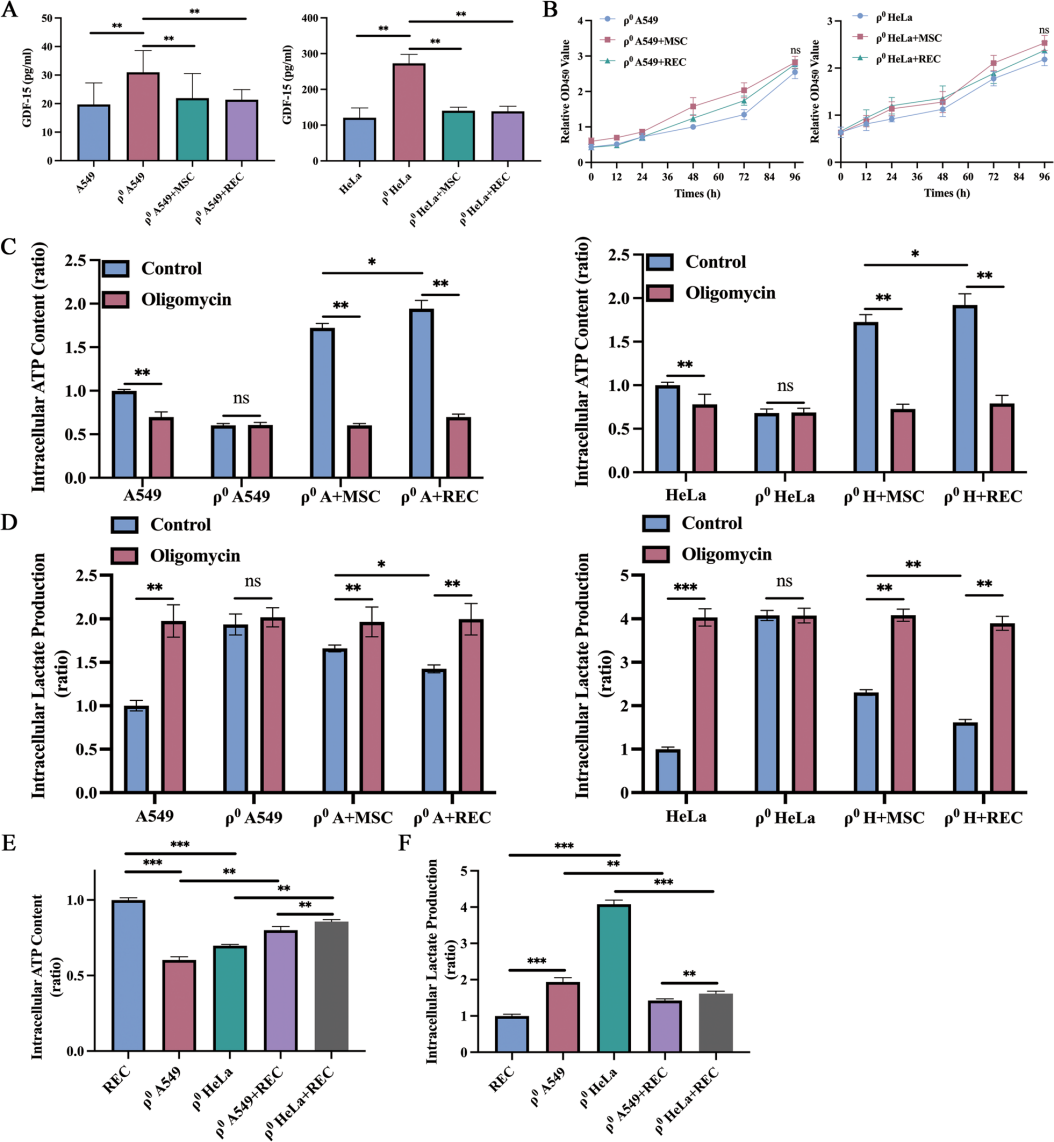
RECs mediated mitochondrial transfer rescued mitochondria-reliant ATP production and shifted metabolism from glycolysis toward OXPHOS. **A** The level of GDF-15 in the different groups of cell culture media. **B** In non-contact co-culture system, CCK-8 was used to detect the proliferation of ρ^0^ A549 and ρ^0^ HeLa cells at different times after co-culture with MSC and REC. **C** Intracellular content of ATP was determined in the different groups with the intervention of Oligomycin. **D** Intracellular content of lactate in the different groups with the intervention of Oligomycin. **E** The intracellular ATP content of different ρ^0^ cell lines was detected. **F** The intracellular lactate content of different ρ^0^ cell lines was detected. All co-culture groups were in a non-contact co-culture system. Data were based on three independent experiments. *p < 0.05, **p < 0.01, ***p < 0.001, ns: no statistical significance between the indicated groups

Several reports have recently suggested that GDF-15 may be an effective marker of mitochondrial damage and thus is helpful in diagnosing mitochondrial diseases [33, 34]. However, the relationship between therapeutic intervention and GDF-15 levels has not been fully determined and reported. We examined the GDF-15 levels in cell culture media of ρ^0^ cells and MSCs/RECs-treated ρ^0^ cells. The average level of GDF-15 in cell culture media was 31.0 ± 6.9 pg/mL in the ρ^0^ A549 group and 272.9 ± 22.8 pg/mL in the ρ^0^ HeLa group, which is significantly high compared to that in the normal cells (A549: 19.7 ± 6.9 pg/mL; HeLa: 120.6 ± 25.3 pg/mL), suggesting the pathologic condition of the ρ^0^ cells. After MSCs and RECs treatment, the GDF-15 levels in ρ^0^ cells were significantly reduced in both cell types (Fig. 10A). We found no significant change in the proliferation of ρ^0^ cells after treatment with MSCs or RECs in the non-contact co-culture system (Fig. 10B).

### RECs significantly increased the bioenergetic productivity

The intracellular ATP level was measured to investigate the bioenergetic productivity of MSCs and REC-rescued ρ^0^ cells. In addition, oligo-responsive ATP suppression was tested to see if the cellular energy source depended on OXPHOS. Co-culture with MSCs or RECs, which had active OXPHOS, demonstrated more than onefold ATP production relative to ρ^0^ cells (Fig. 10C). These results suggested that ρ^0^ cells, after co-culture with MSCs or RECs, mainly produced ATP by OXPHOS. ATP levels in ρ^0^ cells co-cultured with RECs were significantly higher than those in ρ^0^ cells co-cultured with MSCs (Fig. 10C). The amount of intracellular lactate was measured in combination with Olig therapy to indicate that MSCs and REC-donated mitochondria caused a change in metabolic dependence from glycolysis to OXPHOS. MtDNA-deficient ρ^0^ cells displayed a significantly higher basal lactate production than OXPHOS-active cells, such as ρ^0^ A549 + MSC, ρ^0^ A549 + REC, ρ^0^ HeLa + MSC, and ρ^0^ HeLa + REC group cells (Fig. 10D). Lactate levels in ρ^0^ cells co-cultured with RECs were significantly lower than that in ρ^0^ cells co-cultured with MSCs. Treatment with the OXPHOS inhibitor Olig did not alter the lactate levels in ρ^0^ cells, whereas it induced lactate production in each group of cells (Fig. 10D). These findings imply that mitochondrial transfer via MSCs and RECs switched metabolism from glycolysis to OXPHOS and significantly increased bioenergetic production. Also, we compared the differences in the intracellular ATP (Fig. 10E) and lactate content (Fig. 10F) of the two ρ^0^ cells under RECs intervention. We found that the intracellular ATP content of ρ^0^ HeLa cells was significantly higher than that of ρ^0^ A549 cells, and the lactate content was significantly decreased.

### Effect of cell passage on mitochondrial quality in RECs

**Fig. 11:**
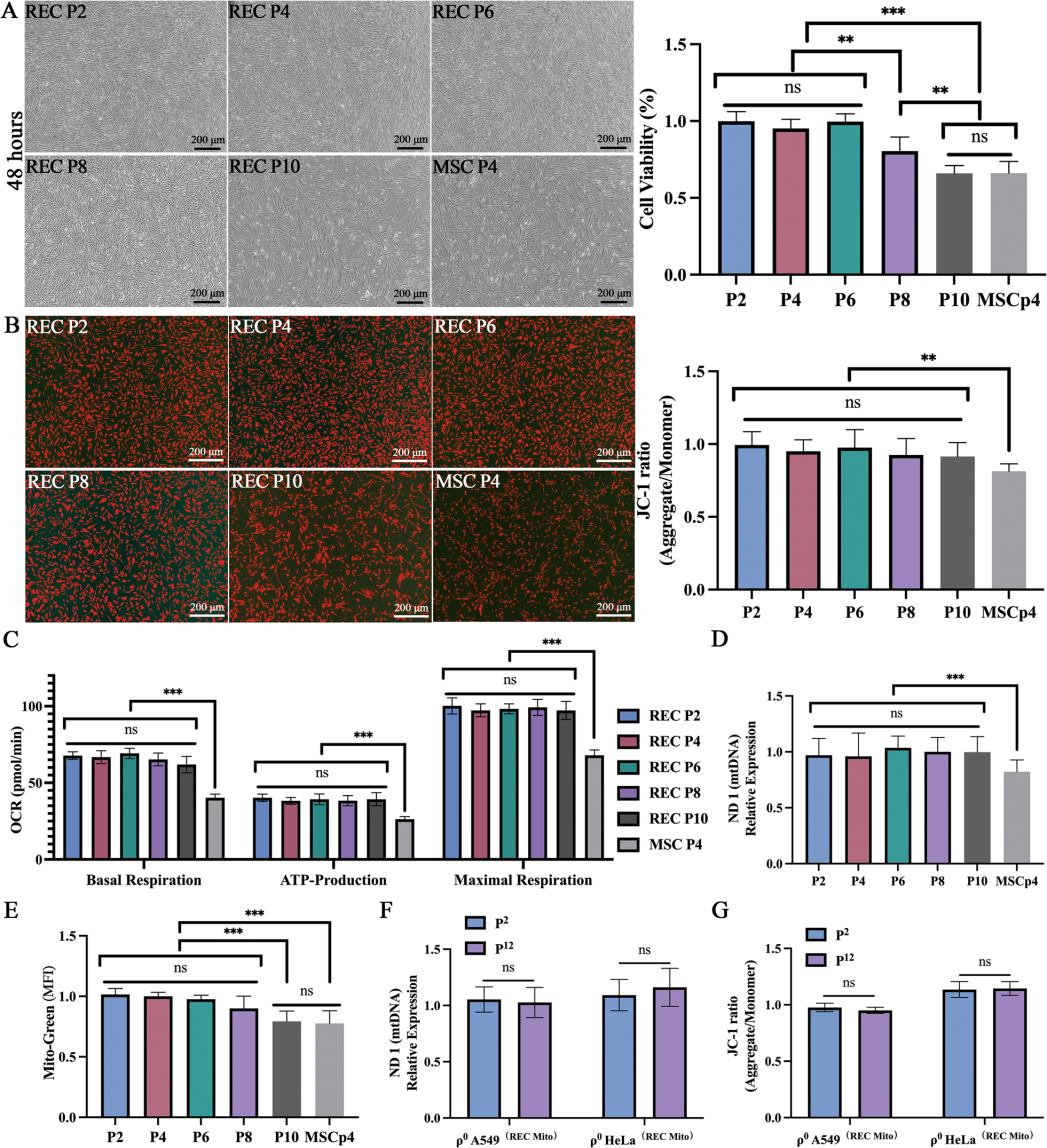
Effect of cell passage frequency on mitochondrial quality in REC. **A** Representative images show the growth state of REC seeded with the same number of cells at different passages after 48 h and the cell viability of REC cells under different passages. **B** Representative fluorescence images showing the mitochondrial membrane potential of REC cells at different passages and the fluorescence intensity ratio of mitochondrial membrane potential levels (JC-1) in different passages. **C** Oxygen consumption rate of REC cells under different passages. **D** mtDNA content of REC cells under different passages. **E** The mean fluorescence intensity (Mito-Green) of REC cells under different passages. **F** RT-qPCR quantitatively analyzes the effect of ρ^0^ cells (receiving REC-donated mitochondria) on mtDNA content in the 2 and 1 Revisiting the environmental Kuznets curve in China: A spatial dynamic panel data approach 2 passages. **G** The fluorescence intensity ratio of mitochondrial membrane potential levels (JC-1) in the 2 and 12 passages ρ^0^ cells (receiving REC-donated mitochondria). Data were based on three independent experiments. *p < 0.05, **p < 0.01, ***p < 0.001, ns: no statistical significance between the indicated groups

To reveal the effect of cell passage rate on mitochondrial quality of RECs, we detected changes in REC at passages 2, 4, 6, 8, and 10, cell proliferation (Fig. 11A), mitochondrial membrane potential (Fig. 11B), oxygen consumption rate, mtDNA content, and mitochondrial content, respectively. We found no significant differences in mitochondrial membrane potential (Fig. 11B), oxygen consumption rate (Fig. 11C), and mtDNA content (Fig. 11D) in RECs of these passages at the same cell number. These indicate that the mitochondrial quality of RECs is guaranteed at least until the 10 passages. But, Fig. 11A showed the growth state of RECs seeded with the same number of cells at different passages after 48 h. It was found that the proliferation rate of RECs decreased significantly from the 8 passages, and the morphology of the cells was more extensive. The number of mitochondria in the 10 passages RECs decreased to a certain extent (Fig. 11E). Therefore, we also recommend using 5–6 passages of RECs. To investigate whether the mitochondrial quality donated by RECs was affected as the passage frequency of ρ^0^ cells increased. We examined the mitochondrial quality of ρ^0^ cells in the 2 and 12 passages. We found no significant differences in mtDNA content and mitochondrial membrane potential of ρ^0^ cells before at least the 12 passages (Fig. 11F, G).

## Discussion

Mitochondrial disorders are a group of multi-system diseases caused by the defects of mtDNA or nuclear DNA, which lead to mitochondria morphology and functional abnormalities. MSCs-mediated mitochondrial transfer has been shown to improve cellular bioenergetics and prevent cell death caused by mitochondrial dysfunction [20, 32]. The ability of RECs in the mitochondrial transfer is unknown. In this study, we discovered a new role for RECs in transferring mitochondria to cells with mitochondrial malfunction. Mitochondria supplied by RECs assisted the recipient cells by supplementing mtDNA, normalizing respiratory function, and improving cellular bioenergetics. These findings support the notion that mitochondrial transfer from RECs to mtDNA-deficient cells might restore normal physiological activities associated with healthy mitochondria.

The human mitochondrial genome can encode several mitochondria-specific proteins, rRNAs, and tRNAs [19, 20]. Since mtDNA is not protected by histones, has a limited rate of self-repair efficiency [1], and is exposed to oxygen radicals generated by oxidative phosphorylation [35], its mutation rate is high. Therefore, interventions aiming at preserving mtDNA stability are required. As a basis, establishing a stable mtDNA-deficient cell model is a prerequisite for this study. Regarding the classical mtDNA-deficient cell model, King et al. [20, 36]. showed that cell lines such as A549, the human alveolar adenocarcinoma cell line, and HeLa, the cell line derived from cervical cancer, could construct stable mtDNA-deficient cell models. Our study found no mtDNA in ρ^0^ cells using the same modeling approach [26], indicating that the modeling was successful. Meanwhile, the changes in the energy metabolism of cancer cells are closely related to abnormal mitochondrial function [5, 17]. Many studies have also shown that malignant tumors may be a kind of energy metabolic disease with respiratory insufficiency caused by mitochondrial dysfunction [37, 38]. Therefore, we established mtDNA-deficient cells with A549 and HeLa cell lines to investigate the role of exogenous functional mitochondria in maintaining mtDNA stability in mtDNA-deficient cells and the biological function of transplanted mitochondria in cancer cells.

MSCs have been shown to transfer mitochondria to other cells under various conditions [10, 20, 22, 39, 40]. Recent research has also revealed that MSCs mitochondrial transfer can restore the mitochondrial function of recipient cells [20, 29] and be a key mechanism for tissue regeneration [9] and repair [41]. TNTs seem to be the first reported, mitochondrial transfer pathway for MSCs [20]. They are spontaneous membranous tubular protrusions that extend from the plasma membrane and allow the transport of various cellular components or organelles [42]. Of all the transport payloads described previously, mitochondria appear to be the most often reported organelle that can be transported unidirectionally or bidirectionally by TNTs. In an in vivo study of acute lung injury, Islam et al. [9]. highlighted the active role of the transmembrane gap junction protein Cx43 in mitochondrial transfer by promoting the formation of TNTs and MVs. The down-regulated expression of Cx43 also reduced the proportion of microtubules in astrocytoma cells [43], suggesting that Cx43 has a role in stabilizing cell membrane microtubules in tumor cells. Therefore, these three transfer pathways have both interaction and uniqueness. In this study, we focused on these three pathways of mitochondrial transfer from RECs to mtDNA-deficient cells compared to that by regular MSCs. Both MSCs and RECs were capable of donating mitochondria to ρ^0^ cells. This transfer is rapid, unidirectional, and is mediated by multiple mechanisms, including MVs, connexin-43 GJs, and TNTs. However, compared to MSCs, we found that the content of TNTs generated by RECs was significantly lower, consistent with previous reports [24]. One probable explanation for this is high migration rate of RECs. The equilibrium response of G-actin incorporation and dissociation balances the synthesis of F-actin. F-actin is a critical component of microfilaments generated by the polymerization of monomeric actin (G-actin). Microfilaments anchor to the cell membrane and are involved in cytoskeletal organization, resulting in successful cell motility. Non-motile cells, on the other hand, frequently collect large bundles of microfilaments known as stress fibers [44]. RECs have high migration, so they produce less TNTs. Through cell inhibition experiments, we also found that compared with MSCs, the mitochondrial transfer rate of RECs was much higher under microtubule/TNTs inhibitors (cytochalasin D). Therefore, TNTs may not be the primary method by which RECs donate mitochondria.

Extracellular MVs are currently recognized as crucial intercellular communication carriers [45]. Exosomes (30–150 nm), microvesicles (50–1500 nm), and apoptotic bodies (> 1000 nm) are the three primary forms and sizes into which they can be separated [45]. Due to their larger diameter, MVs created by cellular plasma membrane blebbing can encompass the whole organelle and have been linked to the transfer of mitochondria and mtDNA [13, 28, 46, 47]. Relevant studies have shown that astrocytes may release MVs mitochondrial particles via CD38-mediated mechanisms that enter neurons after stroke[46]. Therefore, MVs might play an important role in the mitochondrial transfer. In our study, we observed RECs-transferred mitochondrial via MVs in the direct contact co-culture system (Additional file 1: Video S1). TEM results showed that mitochondria in microvesicles were also found in ρ^0^ cells co-cultured with RECs. These suggest that MVs might mediate the mitochondrial transfer mechanism of RECs. Using gap junction (carbenoxelone) and endocytosis/MVs (dynasore) inhibitors, the mitochondrial transfer rate of RECs was significantly reduced compared to that of MSCs. These results indicate that RECs depends more on gap junction and MVs transfer mitochondria than MSCs. And the quantitative analysis of the relative fluorescence intensity of Cx43 protein also showed that RECs produced more Cx43. According to related investigations, MSCs give their functioning mitochondria to alveolar epithelial cells via MVs in a Cx43-dependent way [9]. Additionally, MVs participate in the transmitophagy of stressed MSCs [14] and injured retinal ganglion cells [48], resulting in the self-preservation and reuse of depolarized mitochondria. These findings imply that REC's primary mitochondrial transport mechanism might be Cx43-mediated microvesicle release in the direct contact co-culture system.

In the non-contact co-culture system, we also found cell microvesicles containing mitochondria in ρ^0^ cells co-cultured with RECs. Therefore, MVs may play an important role in the mitochondrial transfer mechanism of RECs in both co-culture systems. In addition, we observed that the mtDNA content of RECs was significantly higher than that of MSCs. Related reports have also shown that as they are limited in size, exosomes that are derived from endosomal cell membranes can only load small proteins, lipids, and RNAs, as well as mtDNA [49]. Therefore, the increased mtDNA content may also be associated with exosomes. Our study also found that the mtDNA content of ρ^0^ cells co-cultured with RECs was significantly higher than that of ρ^0^ cells co-cultured with MSCs. Therefore, It is speculated that the possible pathway of mtDNA transfer by RECs includes mitochondrial transfer and mtDNA carried by exosomes. And exosomes may play an important role in the mitochondrial transfer pathway. Exosomes of RECs with typical double-layered membrane vesicles were observed under TEM. Zhao et al. also found that extracellular vesicles derived from MSCs can reduce mitochondrial damage and inflammation by stabilizing mitochondrial DNA [50]. Therefore, extracellular vesicles are lieky to be one of the important mechanisms regulating mitochondrial transfer in RECs cells.

MSCs have been suggested to cure the ATP deficit and OCR by providing mtDNA to ρ^0^ cells [20, 32]. However, additional biological advancements are required in the use of regenerative medicine to obtain excellent clinical safety and efficacy. High-quality MSCs are required for clinical use. The International Society for Cell Therapy (ISCT) has established a standard for human MSCs [51, 52]. The use of RECs represented a significant advantage of this study because the cell source produced high-quality MSCs with minimal inter-batch variation and showed superior properties in terms of cell proliferation, cell size uniformity, and surface antigen expression compared to that of MSCs [24, 25]. In addition, in this study, we found that RECs could deliver more mitochondria to ρ^0^ cells than MSCs in both contact and non-contact co-culture systems, and RECs was superior to MSCs in terms of mitochondrial content, mitochondrial membrane potential, and OCR. It is worth mentioning that RECs itself was superior to MSCs in mitochondrial content, mtDNA content, mitochondrial membrane potential, and OCR. This is also a strong guarantee that RECs can better restore mitochondrial function in mtDNA-deficient cells. Intriguingly, according to Ukeba et al. research, using ultra-purified MSCs in the form of RECs and situ forming gel promotes lumbar intervertebral disc (IVD) regeneration following discectomy in a sheep model with severe IVD degeneration without causing neoplastic alterations [25]. The fundamental issue regarding the safety of such therapeutic modalities is whether malignancies could form following stem cell transplantation. Our results showed that RECs did not affect the proliferation of ρ^0^ cells in either direct contact or non-contact co-culture system. The length of time that RECs can sustain the increase in mitochondrial function is crucial for the development of a REC-based treatment method, as intracellular biological systems may destroy the transplanted mitochondria or mtDNA. One method of mitochondrial transfer has been found to enter cells with mitochondrial failure and survive for at least 21 days by delivering isolated peptide-modified mitochondria [53]. Our findings reveal that mtDNA might can persist, with mitochondria-related functions maintained for at least 12 passage, implying that it is possible to retain the therapeutic impact of mitochondria following mitochondrial transfer from RECs. Despite the advantages observed in RECs as compared to MSCs, further studies are needed to clarify whether RECs is superior in the treatment of mitochondrial dysfunction.

This study has several limitations. First, our observations were based entirely on in vitro experiments. The pathophysiological relevance of our findings should be confirmed in vivo, using rodent models and clinical studies. Second, although different mitochondrial transfer pathways have been revealed in this study, such as TNTs [8, 10, 20], Cx43-formed GJs [9, 11, 12], and MVs secretion [13, 14], the specific mechanism of mitochondrial transfer needs to be elucidated.

## Conclusion

We have provided an alternative, efficient, homogenized, REC-based therapeutic strategy to supplement healthy mitochondria to rescue bioenergetic demands and OXPHOS-dependent processes. However, REC's efficiency and therapeutic effect in mitochondrial transfer to other cells or animal models of mitochondrial disease require further investigation.

## Supplementary Information

**Additional file 1**. **Video 1**: MITO-Green labeled RECs were co-cultured with DID labeled ρ^0^ HeLa cells. Time-lapse video shows the mitochondria in microvesicles (MVs) were moving from RECs to ρ^0^ HeLa cells. Red arrow shows this process.

**Additional file 2**. **Figure S1**: ND1, COX1, HVR2, CH1 original uncropped images. **Figure S2:** Repeat the experiment with the original uncropped images.

## Data Availability

The datasets used and/or analyzed during the current study are available from the corresponding author upon reasonable request.

## Abbreviation

MSCs: Mesenchymal stem cells
mtDNA: Mitochondrial DNA
nDNA: Nuclear DNA
RECs: Rapidly expanding clones
TNTs: Tunneling nanotubes
FCM: Flow cytometry
OCR: Oxygen consumption rate
OXPHOS: Oxidative phosphorylation
ND1: NADH dehydrogenase 1
COX-1: Cytochrome oxidase-1
HVR2: Hypervariant, and region 2
CCK-8: Cell counting kit-8
ROS: Reactive oxygen species
mtROS: Mitochondrial ROS
EVs: Extracellular vesicles
GJs: Gap junctions
TNTs: Tunneling nanotubes
CBX: Carbenoxelone
Cyto-D: Cytochalasin D
CFSE: Carboxyfluorescein succinimidyl ester
ISCT: International Society for Cell Therapy
