# Supplementary material for: Highly-purified rapidly expanding clones, RECs, are superior for functional-mitochondrial transfer
Source: Stem Cell Res Ther. 2023 Mar 16;14:40. doi: 10.1186/s13287-023-03274-y (PMC10022310; doi:10.1186/s13287-023-03274-y)

**Supplementary Figures**

**Figure1**

**Original uncropped images**

**
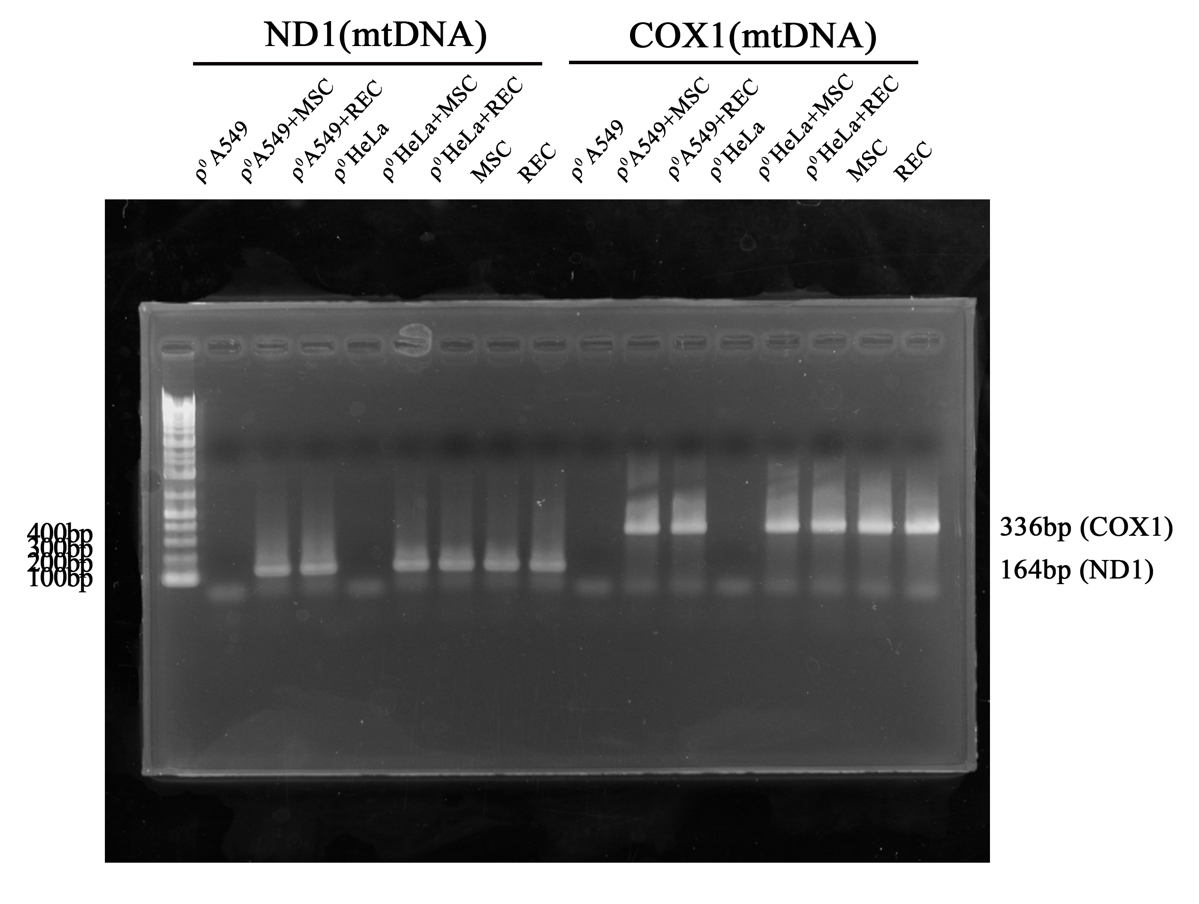
**

**
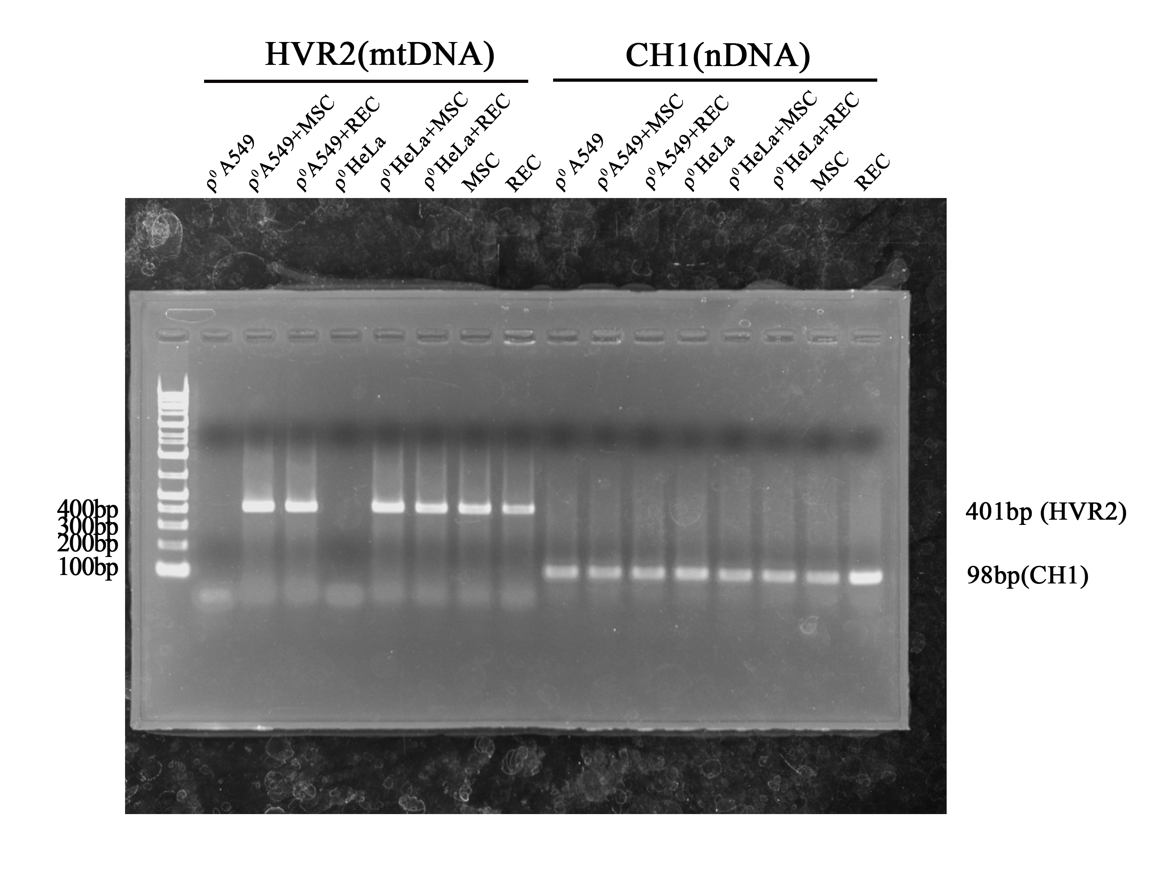
**

**Figure2**

**Repeat the experiment with the original uncropped images**


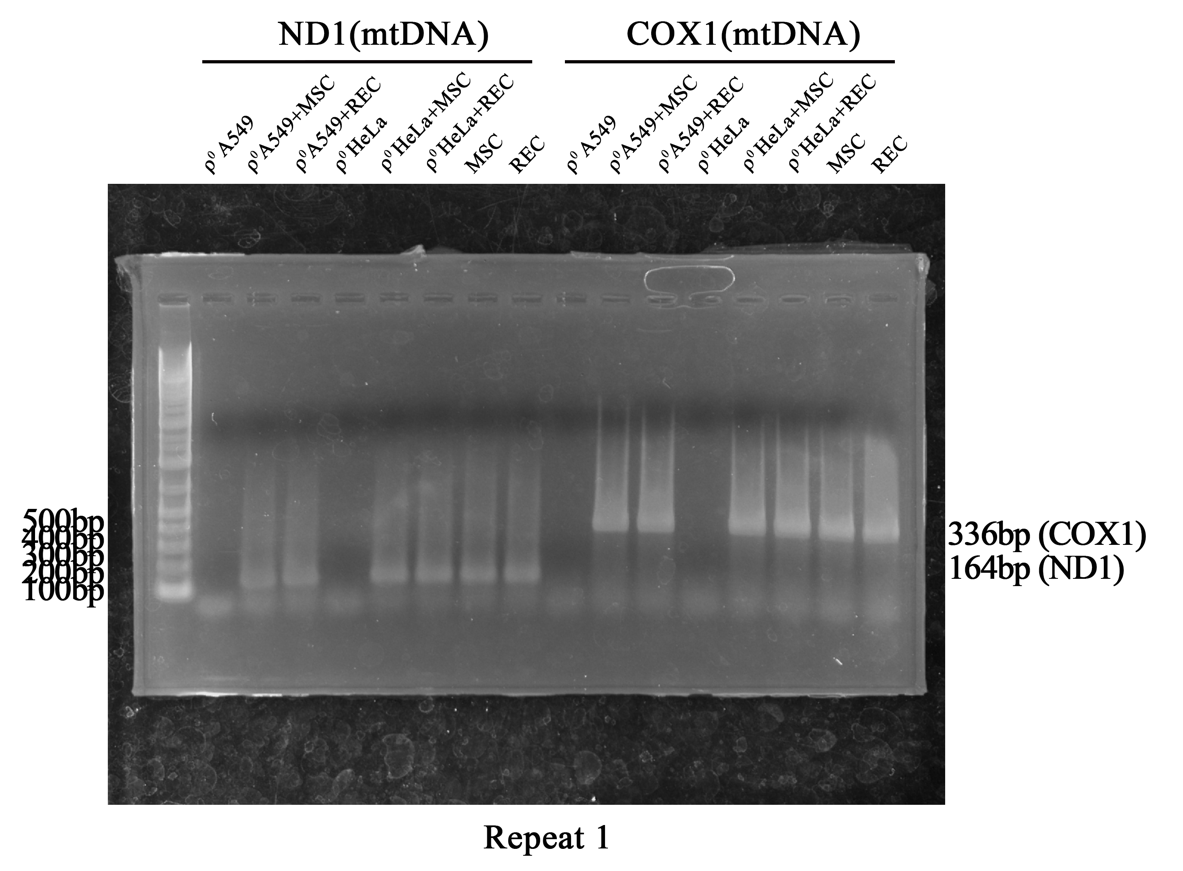


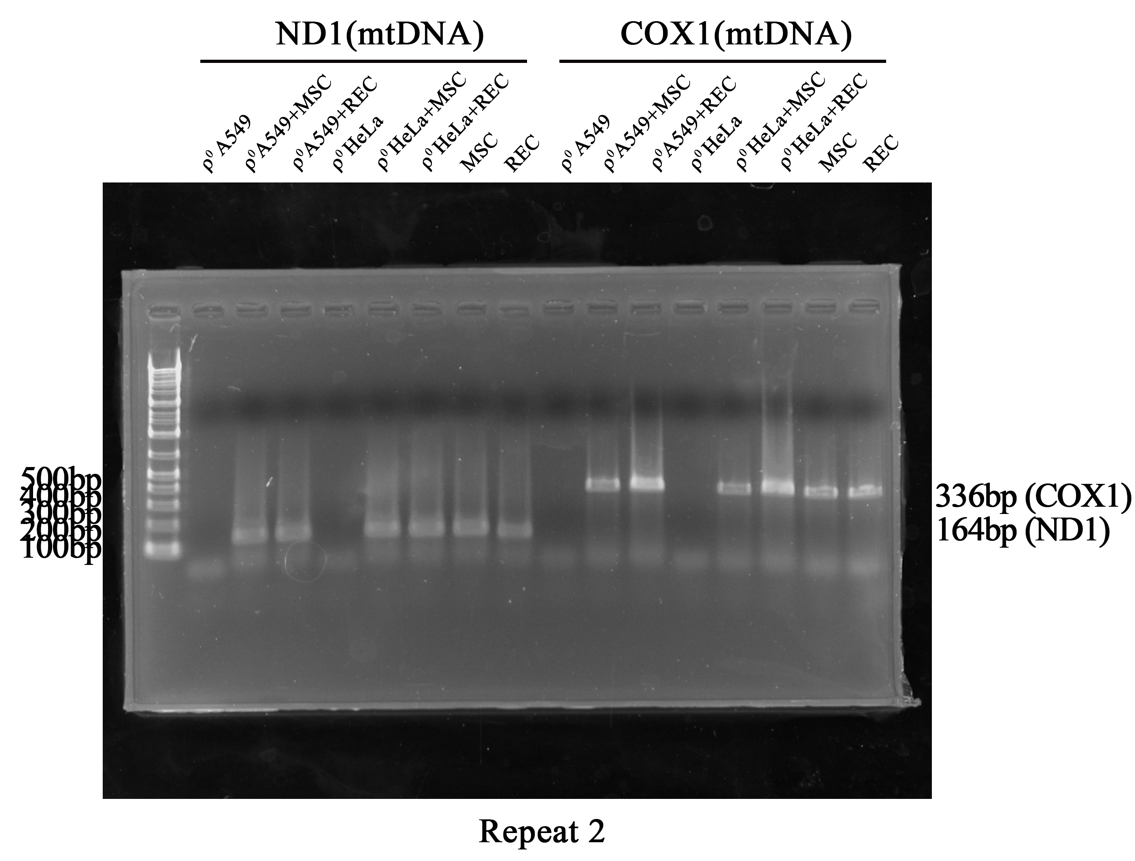


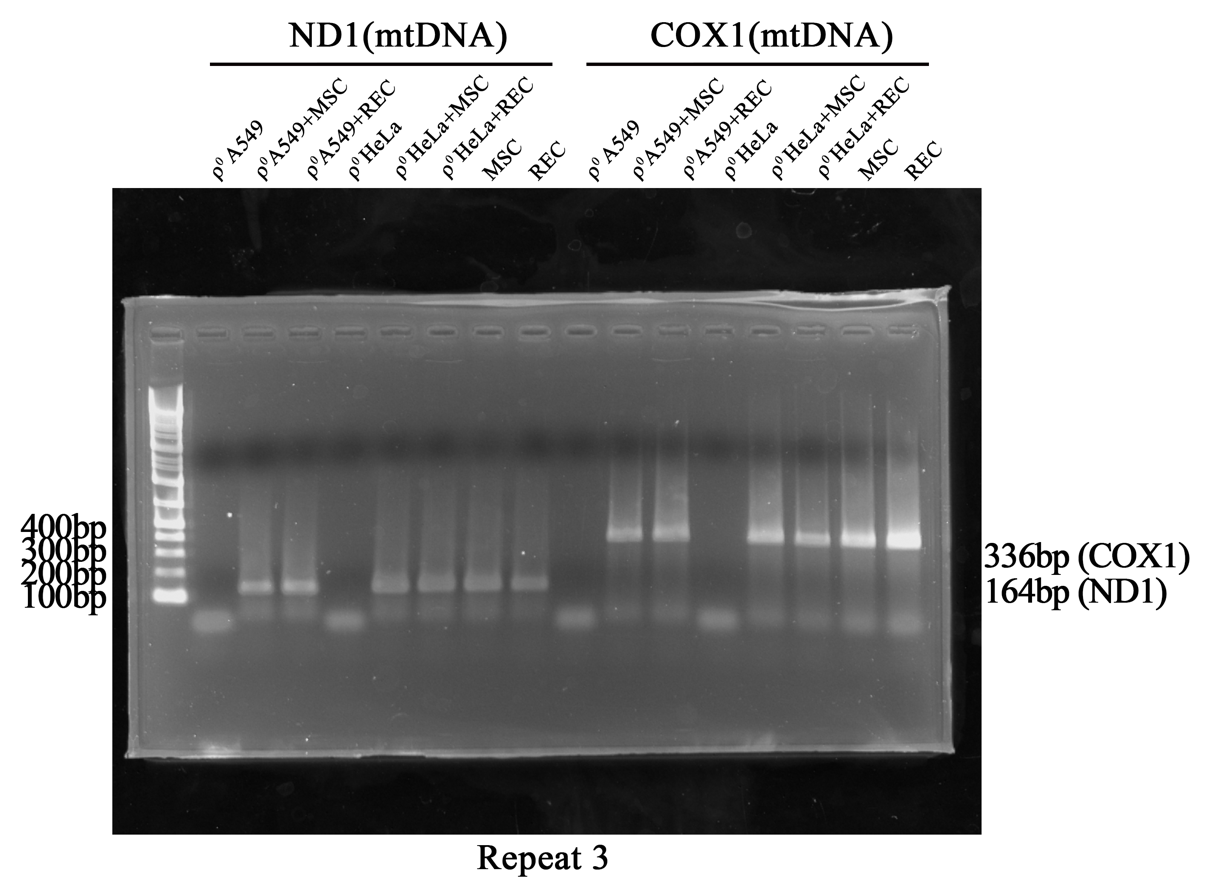


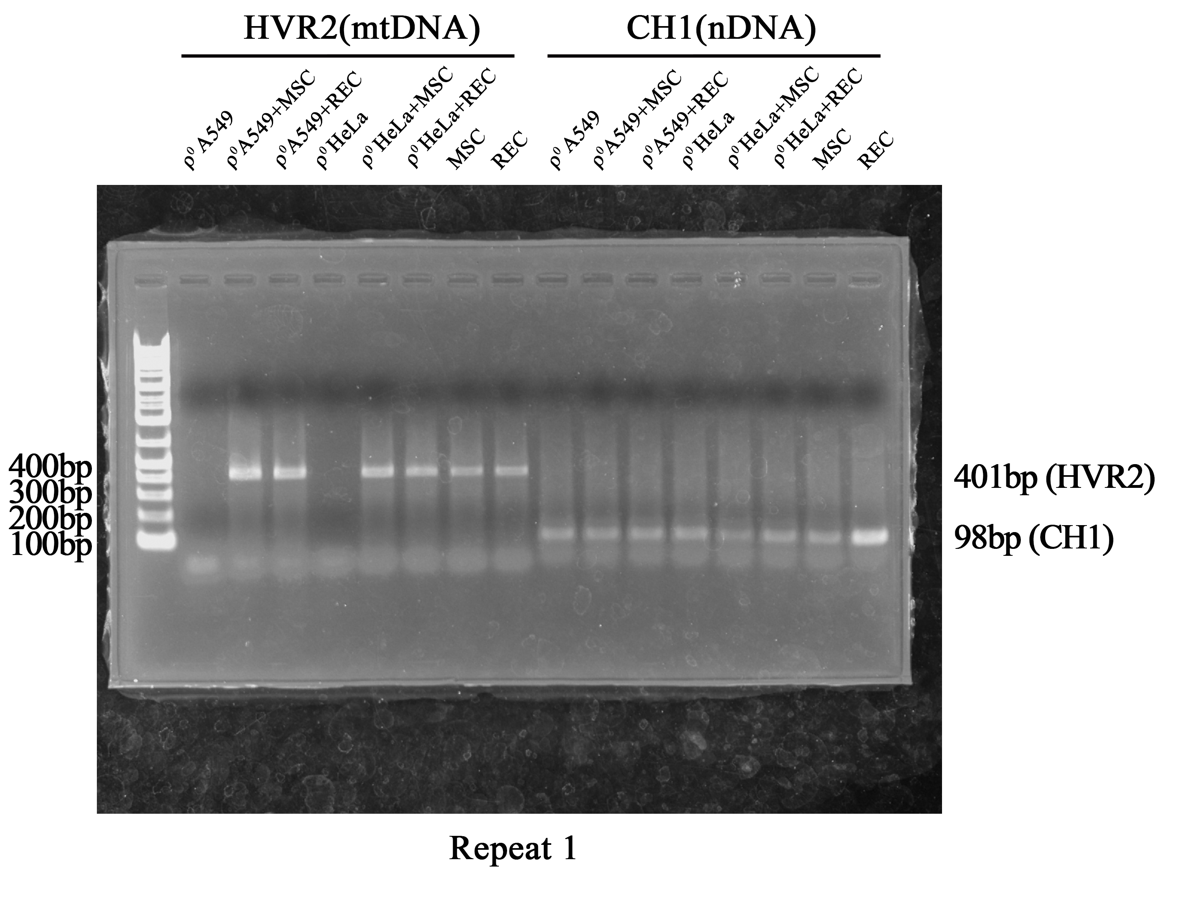


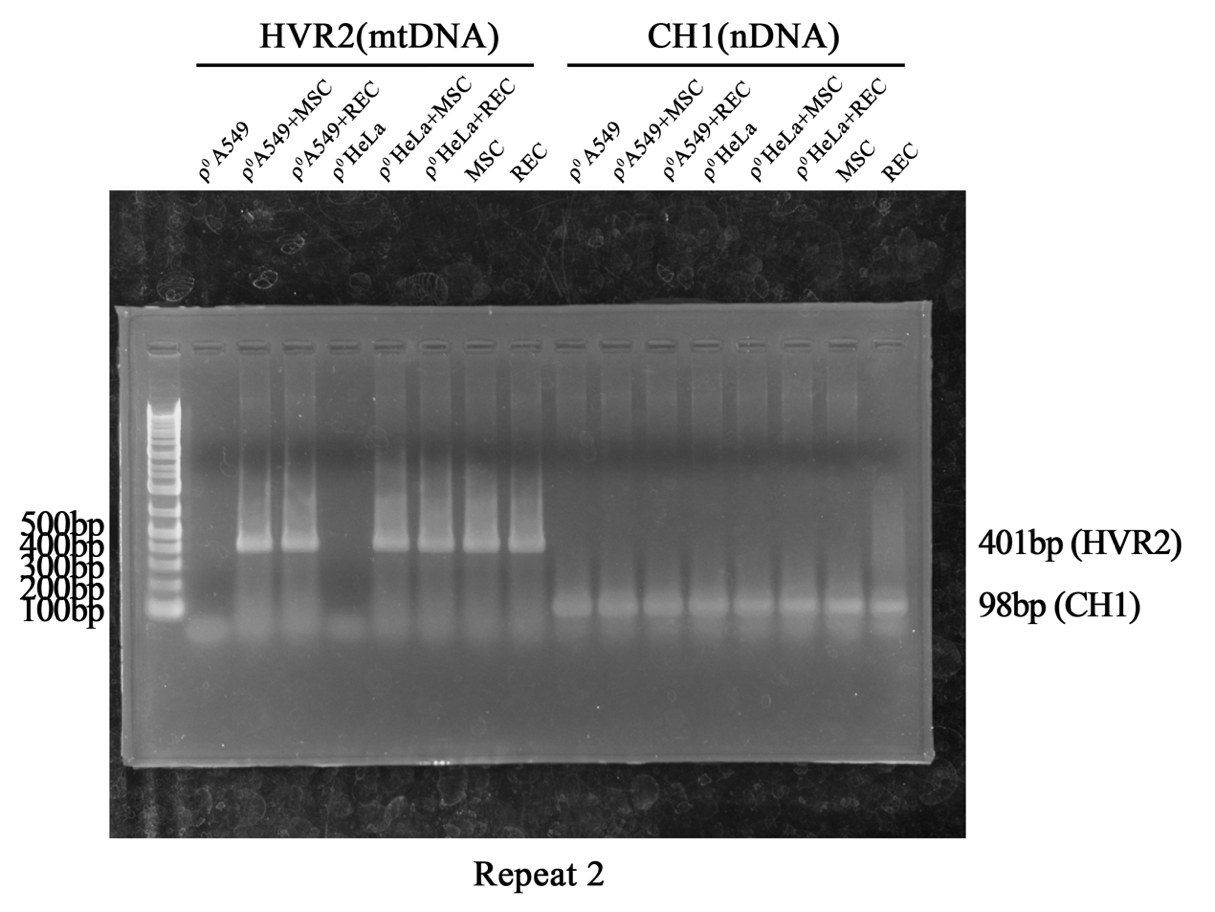


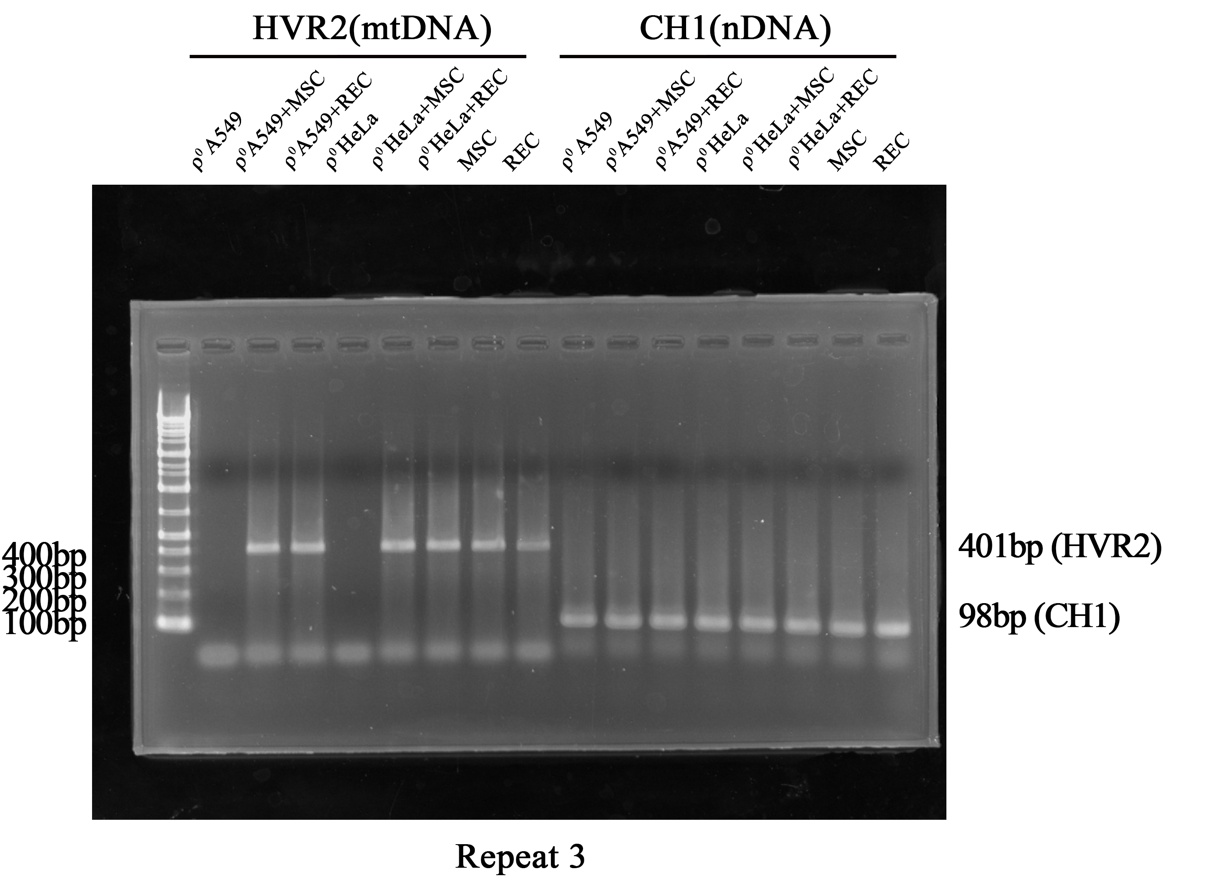

Supplement: Supplementary file 2 — Additional file 2. Figure S1: ND1, COX1, HVR2, CH1 original uncropped images. Figure S2: Repeat the experiment with the original uncropped images. [file 13287_2023_3274_MOESM2_ESM.docx]
